# AI-driven translations for kidney transplant equity in Hispanic populations

**DOI:** 10.1038/s41598-024-59237-7

**Published:** 2024-04-12

**Authors:** Oscar A. Garcia Valencia, Charat Thongprayoon, Caroline C. Jadlowiec, Shennen A. Mao, Napat Leeaphorn, Pooja Budhiraja, Iasmina M. Craici, Maria L. Gonzalez Suarez, Wisit Cheungpasitporn

**Affiliations:** 1 Division of Nephrology and Hypertension, Department of Medicine, Mayo Clinic, Rochester, MN USA; 2 Division of Transplant Surgery, Mayo Clinic, Phoenix, AZ USA; 3 Division of Transplant Surgery, Department of Transplantation, Mayo Clinic, Jacksonville, FL USA; 4 Department of Transplant, Mayo Clinic, Jacksonville, USA

**Keywords:** Artificial intelligence, Large language models, Health equity, Kidney transplant information, Spanish translation, Cultural sensitivity, ChatGPT, Evolution of language, Computational biology and bioinformatics, Health care, Nephrology, Engineering, Mathematics and computing

## Abstract

Health equity and accessing Spanish kidney transplant information continues being a substantial challenge facing the Hispanic community. This study evaluated ChatGPT’s capabilities in translating 54 English kidney transplant frequently asked questions (FAQs) into Spanish using two versions of the AI model, GPT-3.5 and GPT-4.0. The FAQs included 19 from Organ Procurement and Transplantation Network (OPTN), 15 from National Health Service (NHS), and 20 from National Kidney Foundation (NKF). Two native Spanish-speaking nephrologists, both of whom are of Mexican heritage, scored the translations for linguistic accuracy and cultural sensitivity tailored to Hispanics using a 1–5 rubric. The inter-rater reliability of the evaluators, measured by Cohen’s Kappa, was 0.85. Overall linguistic accuracy was 4.89 ± 0.31 for GPT-3.5 versus 4.94 ± 0.23 for GPT-4.0 (non-significant p = 0.23). Both versions scored 4.96 ± 0.19 in cultural sensitivity (p = 1.00). By source, GPT-3.5 linguistic accuracy was 4.84 ± 0.37 (OPTN), 4.93 ± 0.26 (NHS), 4.90 ± 0.31 (NKF). GPT-4.0 scored 4.95 ± 0.23 (OPTN), 4.93 ± 0.26 (NHS), 4.95 ± 0.22 (NKF). For cultural sensitivity, GPT-3.5 scored 4.95 ± 0.23 (OPTN), 4.93 ± 0.26 (NHS), 5.00 ± 0.00 (NKF), while GPT-4.0 scored 5.00 ± 0.00 (OPTN), 5.00 ± 0.00 (NHS), 4.90 ± 0.31 (NKF). These high linguistic and cultural sensitivity scores demonstrate Chat GPT effectively translated the English FAQs into Spanish across systems. The findings suggest Chat GPT’s potential to promote health equity by improving Spanish access to essential kidney transplant information. Additional research should evaluate its medical translation capabilities across diverse contexts/languages. These English-to-Spanish translations may increase access to vital transplant information for underserved Spanish-speaking Hispanic patients.

## Introduction

The concept of health equity involves providing every individual with a fair and just opportunity to attain their highest level of health^1^. Unfortunately, disparities in healthcare access and the distribution of medical information continue to be significant barriers^2^. For the Hispanic community, particularly those who primarily speak Spanish, these barriers are often compounded by linguistic challenges, limiting their access to essential healthcare information^3–6^. A recent study examined trends in poor health indicators among Black and Hispanic middle-aged and older adults in the United States from 1999 to 2018^7^. The study found that, while Hispanics showed overall improvements in physical inactivity and perceived poor health, they experienced deterioration in hypertension and diabetes rates. Notably, the study reported no significant change in the Hispanic-White gap for kidney disease over the 20-year period, indicating that the disparity in this specific condition did not improve^7^. In the context of kidney transplantation, where understanding complex medical information is crucial, this language barrier presents a substantial obstacle^8,9^. The Hispanic population is disproportionately affected by kidney diseases, including higher prevalence rates of conditions leading to kidney failure. According to epidemiological studies, Hispanics are more likely to develop end-stage kidney disease (ESKD) compared to non-Hispanic whites^10–12^.

Additionally, they face longer waiting times for kidney transplants and lower rates of referral for transplant evaluations^9,13^. These disparities can be attributed to several factors, including language barriers that impede effective communication between healthcare providers and patients, leading to misunderstandings, missed appointments, and incomplete or inaccurate medical documentation. Moreover, the lack of culturally and linguistically appropriate health information contributes to a lower level of health literacy among this population, further complicating their navigation through the transplant referral and evaluation process^3–6^.

The provision of culturally appropriate health information is crucial in managing and treating chronic conditions like kidney disease. Culturally sensitive information takes into account not just the language but also the cultural beliefs, practices, and values of a community^14–16^. This approach is particularly important in the Hispanic community, where cultural nuances play a vital role in health-related decision-making. Additionally, Language barriers can significantly impact the quality of healthcare received by non-English speaking patients^17,18^. In the United States, a considerable portion of the Hispanic population has limited English proficiency, making it challenging for them to access and understand health information in English^18,19^. This gap is not just a matter of translation but involves conveying complex medical concepts in a linguistically and culturally appropriate manner.

Artificial intelligence, particularly advanced language models like Chat GPT 3.5 and 4.0, presents an innovative approach to addressing the challenges of language barriers in healthcare^20–28^. These AI models hold the potential to accurately and sensitively translate complex medical information, thereby making it accessible to a wider audience^29–36^. In the specific context of kidney transplantation, where the necessity for detailed and accurate information is critical, the role of AI-driven translations could be transformative, offering a significant advancement in how medical information is communicated to non-English speaking populations.

The primary objective of this study is to evaluate the effectiveness of Chat GPT 3.5 and 4.0 in translating kidney transplantation-related FAQs from English to Spanish, tailored for the Hispanic community. The study focuses on the accuracy and cultural sensitivity of these translations, assessing whether these AI tools can provide reliable, comprehensible, and culturally appropriate medical information. By doing so, the study seeks to determine the potential of AI in improving health information accessibility and contributing to health equity for Spanish-speaking Hispanics.

## Materials and methods

### Data collection

This study was conducted to perform English-to-Spanish translation of 54 frequently asked questions (FAQs) regarding kidney transplantation. The FAQs were selected to comprehensively represent the relevant topics to patients considering or undergoing kidney transplantations. The FAQs was obtained from (1) Organ Procurement and Transplantation Network (OPTN)^37^; 19 questions focusing on eligibility criteria, waitlist process, and post-transplant care, (2) National Health Service (NHS)^38^; 15 questions focusing on patient preparation for kidney transplantation, surgical procedures, and post-transplant care, (3) National Kidney Foundation^39^; 20 questions focusing on long-term management, lifestyle consideration, and support resources for kidney transplant recipients (Online supplementary data). This study is exempt from Ethics Committee or Institutional Review Board approval, as it neither involves human nor animal subjects, nor does it encompass patient information or identifiable personal data.

### AI language model usage

The translation process utilized ChatGPT versions 3.5 and 4.0^40^. These AI chatbots were chosen for their advanced natural language processing capabilities^41,42^, which include the ability to understand context, generate coherent and contextually appropriate text, and maintain consistency in translations. Each selected FAQ was input into the AI chatbot in its English version, and the models then provided Spanish translations. This process was conducted individually for each question to ensure that each translation was contextually accurate. The AI chatbots were configured to optimize for translation accuracy and cultural relevance, focusing on nuances that would make the translations suitable for the Hispanic community. The study was conducted in December 2023.

### Systematic evaluation of translations

Each translation was evaluated using a detailed rubric scale ranging from 1 to 5 (Online supplementary data), when 1 represents a lower or poor performance and 5 indicates a higher or excellent performance^43^. The rubric scale was designed to assess two key aspects:

- Linguistic accuracy: This criterion evaluated the grammatical correctness, appropriate use of vocabulary, and syntactic integrity of the translations. Translations were examined for their clarity, readability, and technical precision in medical terminology.
- Cultural sensitivity: This measure assessed the extent to which translations respected and incorporated cultural nuances, idiomatic expressions, and contextually relevant information for the Hispanic community. This aspect was crucial to ensure that the translations were not only linguistically accurate but also culturally resonant and sensitive to the needs of the target audience.

Two nephrologists of Mexican heritage, fluent in Spanish, O.A.G.V. and M.G.S., meticulously evaluated the translations for accuracy and cultural relevance using a 1–5 scale. The evaluation process totaled approximately 40 h, with each expert contributing around 20 h. They began with O.A.G.V.’s initial assessments, which M.G.S. reviewed and confirmed, and any differences were resolved through consensus. The inter-rater reliability of the evaluators, measured by Cohen’s Kappa, was 0.85, indicating a high level of agreement and supporting the reliability and credibility of the findings.

### Statistical analysis

The mean scores for linguistic accuracy and culture sensitivity were summarized as mean ± standard deviation (SD). The score was compared between GPT-3.5 and 4.0 using paired-*t* test. The score was compared across three question sources using analysis of variance (ANOVA) test. The two-tailed p-value less than 0.05 was considered statistically significant. Statistical analyses were performed using JMP statistical software (version 17, SAS Institute, Cary, NC).

## Results

The score for linguistic accuracy and cultural sensitivity of GPT-3.5 and GPT-4.0 for individual FAQs were shown in Table S1. The mean linguistic accuracy score was 4.89 ± 0.31 for GPT-3.5 and 4.94 ± 0.23. There was no significant difference in mean linguistic accuracy score between GPT-3.5 and 4.0 in all questions (p = 0.26) as well as when stratified by FAQ sources. The mean linguistic accuracy score was comparable across three FAQ sources for GPT-3.5 (4.84 ± 0.37 vs. 4.93 ± 0.26 vs. 4.90 ± 0.31 for FAQs from OPTN, NHS, and NKF respectively; p = 0.70) and GPT-4.0 (4.95 ± 0.23 vs. 4.93 ± 0.26 vs. 4.95 ± 0.22 for FAQs from OPTN, NHS, and NKF respectively; p = 0.98) (Table 1).

**Table 1:** The mean score for linguistic accuracy and culture sensitivity of GPT-3.5 and 4.0

Question	Linguistic accuracy			Culture sensitivity		
	GPT-3.5	GPT-4.0	p-value*	GPT-3.5	GPT-4.0	p-value*
All	4.89 ± 0.31	4.94 ± 0.23	0.26	4.96 ± 0.19	4.96 ± 0.19	1.00
OPTN	4.84 ± 0.37	4.95 ± 0.23	0.16	4.95 ± 0.23	5.00 ± 0.00	0.33
NHS	4.93 ± 0.26	4.93 ± 0.26	1.00	4.93 ± 0.26	5.00 ± 0.00	0.33
NKF	4.90 ± 0.31	4.95 ± 0.22	0.58	5.00 ± 0.00	4.90 ± 0.31	0.16
p-value^#^	0.70	0.98		0.55	0.18	

*p-value between GPT-3.5 and 4.0.

^#^p-value between question source.

**Figure 1:**
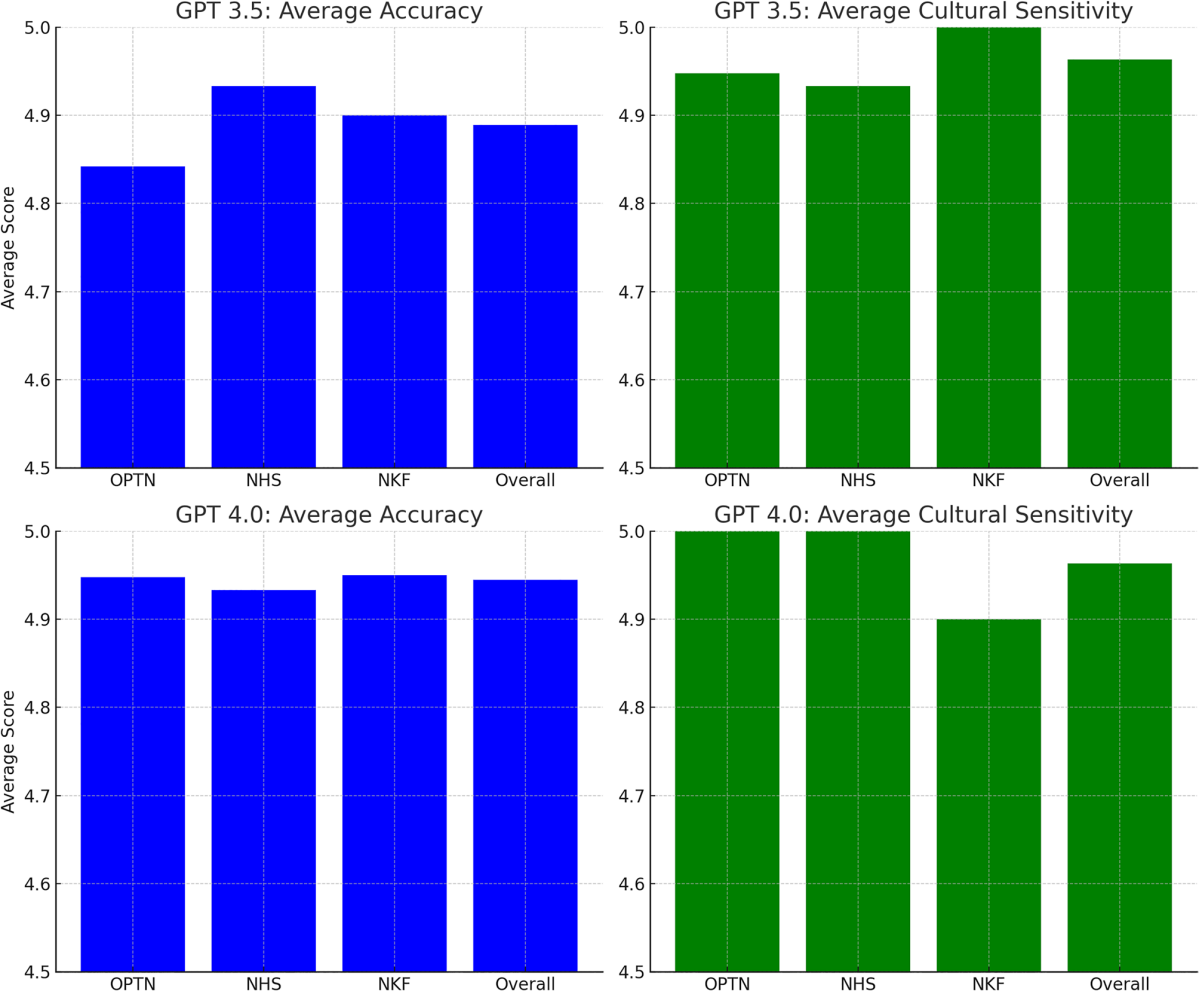
Comparative analysis of average accuracy and cultural sensitivity in AI-generated translations of kidney transplant information. Top panel: (Left) GPT 3.5: average accuracy across different organizations (OPTN, NHS, NKF) and overall score. (Right) GPT 3.5: average cultural sensitivity across different organizations (OPTN, NHS, NKF) and overall score. Bottom panel: (Left) GPT 4.0: average accuracy across different organizations (OPTN, NHS, NKF) and overall score. (Right) GPT 4.0: average cultural sensitivity across different organizations (OPTN, NHS, NKF) and overall score.

The mean culture sensitivity score was 4.96 ± 0.19 for GPT-3.5 and 4.96 ± 0.19 for GPT-4.0. There was no significant difference in mean culture sensitivity score between GPT-3.5 and 4.0 in all questions (p = 1.00) as well as when stratified by FAQ sources. The mean culture sensitivity score was comparable across three FAQ sources for GPT-3.5 (4.95 ± 0.23 vs. 4.93 ± 0.26 vs. 5.00 ± 0.00 for FAQs from OPTN, NHS, and NKF respectively; p = 0.55) and GPT-4.0 (5.00 ± 0.00 vs. 5.00 ± 0.00 vs. 4.90 ± 0.31 for FAQs from OPTN, NHS, and NKF respectively; p = 0.18) (Fig. 1).

## Discussion

The study meticulously evaluated the translation capabilities of ChatGPT 3.5 and 4.0, focusing on translating kidney transplantation FAQs for the Hispanic community. The main results indicate that both versions achieved high levels of accuracy and cultural sensitivity, with ChatGPT 4.0 slightly outperforming 3.5 in terms of accuracy. Specifically, ChatGPT 3.5 demonstrated exceptional cultural sensitivity, especially in the NKF subgroup, while ChatGPT 4.0 consistently scored perfect accuracy across all questions. The study’s results are especially significant in the context of health equity. By offering accurate and culturally sensitive translations, AI models like ChatGPT can play a crucial role in leveling the informational playing field for non-English-speaking communities. This is particularly important for Hispanics affected by kidney diseases, who often encounter linguistic hurdles in accessing vital health information^44–46^. The ability of ChatGPT to provide translations that are not only linguistically accurate but also culturally resonant is key to its effectiveness as a tool for disseminating medical information.

While both versions demonstrated high accuracy and cultural sensitivity, it is noteworthy that ChatGPT 3.5 had occasional lower scores in either accuracy or cultural sensitivity in specific questions. This suggests that while the model is highly effective, there is room for improvement, particularly in handling certain nuances that require deeper cultural understanding. In contrast, ChatGPT 4.0’s consistent scoring of 5 in accuracy for all questions reflects advancements in AI technology, although it too faced challenges in cultural sensitivity in a few instances. The effectiveness of AI in translation is not solely dependent on linguistic accuracy but also on its ability to resonate culturally with the intended audience. This is particularly crucial in healthcare, where the cultural context can significantly impact how information is received and acted upon^47–49^.

Comparing this study’s findings with previous research in AI-driven language translation in healthcare, it’s evident that there have been significant advancements^50–54^. Earlier research often pointed out the limitations of AI models in grasping the complexities of language and cultural context, particularly in medical translations where both accuracy and sensitivity are crucial. These models typically struggled to maintain a balance between literal accuracy and the deeper layers of cultural context, resulting in translations that were technically correct but often lacked relevance and appropriateness in a real-world setting. In contrast, this study highlights a significant progress with ChatGPT 3.5 and 4.0, illustrating their improved ability to not only translate complex medical information accurately but also to consider cultural appropriateness in these translations. This progress signifies a move towards more sophisticated AI models that are linguistically adept and more in tune with the cultural and contextual aspects of language, meeting the practical needs of diverse patient groups, like those seeking information on kidney transplantation.

The study, while pivotal in evaluating the translation capabilities of ChatGPT 3.5 and 4.0 for kidney transplantation FAQs in Spanish for the Hispanic community, presents certain limitations that shape the scope and applicability of its findings. Its focus is narrowly tailored to a specific medical context and a particular linguistic group, which may not encompass the varied complexities of other medical domains or cater to different cultural backgrounds. The reliance on human evaluators introduces an element of subjectivity in assessing translation accuracy and cultural sensitivity, potentially affecting the consistency of the results. Furthermore, the study’s constraint to only two AI models limits a broader comparative analysis across the spectrum of available AI translation technologies. Future research, therefore, should aim to broaden the scope to include diverse medical topics and languages, extend evaluations to a wider range of AI models, and incorporate more objective assessment methods. Such expansion and refinement in research approach would not only enhance the generalizability of the findings but also deepen the understanding of AI’s potential in overcoming language barriers in global healthcare contexts.

In addition, in future research, the exploration of AI-driven translation tools like ChatGPT 3.5 and 4.0 in real clinical practice represents a critical area for advancement, especially in the context of kidney transplantation and health equity. These studies should focus on evaluating the impact of AI translations on patient outcomes, understanding, and engagement in their healthcare journey. Integration with healthcare systems, including electronic health records and patient portals, is also essential to assess the efficiency and effectiveness of AI tools in a clinical setting. Feedback from healthcare providers will be invaluable, offering insights into the practical utility, accuracy, and cultural appropriateness of these translations in enhancing patient care. Additionally, longitudinal studies observing the long-term effects of AI translation tools, their cost-effectiveness, and comparative analyses with traditional translation methods will provide a comprehensive understanding of the role of AI in reducing healthcare disparities. Such research is pivotal in determining the full potential of AI in improving communication and fostering health equity, particularly for linguistically diverse populations in need of specialized medical care like kidney transplantation.

## Conclusions

This study demonstrates the significant potential of advanced AI models like ChatGPT 3.5 and 4.0 in bridging language gaps in the healthcare sector. By providing high-quality translations that are both accurate and culturally sensitive, these tools can greatly enhance the accessibility of medical information, particularly for underserved non-English-speaking populations. As AI technology continues to evolve, its role in supporting health equity and improving patient outcomes across diverse communities becomes increasingly vital.

### Supplementary Information

Supplementary Information.

## Data Availability

The data underlying this article will be shared on reasonable request to the corresponding author.

## References

[1] Braveman P (2006). Health disparities and health equity: Concepts and measurement. Annu. Rev. Public Health.

[2] Braveman PA (2011). Health disparities and health equity: The issue is justice. Am. J. Public Health.

[3] Velasco-Mondragon E, Jimenez A, Palladino-Davis AG, Davis D, Escamilla-Cejudo JA (2016). Hispanic health in the USA: A scoping review of the literature. Public Health Rev..

[4] Pérez-Stable EJ, Nápoles-Springer A, Miramontes JM (1997). The effects of ethnicity and language on medical outcomes of patients with hypertension or diabetes. Med. Care.

[5] Garbers S, Chiasson MA (2004). Inadequate functional health literacy in Spanish as a barrier to cervical cancer screening among immigrant Latinas in New York City. Prev. Chronic Dis..

[6] Timmins CL (2002). The impact of language barriers on the health care of Latinos in the United States: A review of the literature and guidelines for practice. J. Midwifery Women’s Health.

[7] Odlum M (2020). Trends in poor health indicators among Black and Hispanic middle-aged and older adults in the United States, 1999–2018. JAMA Netw. Open.

[8] Pande M, Grafals M, Rizzolo K, Pomfret E, Kendrick J (2022). Reducing disparities in kidney transplantation for Spanish-speaking patients through creation of a dedicated center. BMC Nephrol..

[9] Gordon EJ (2015). Hispanic/Latino disparities in living donor kidney transplantation: Role of a culturally competent transplant program. Transplant. Direct.

[10] Benabe JE, Rios EV (2004). Kidney disease in the Hispanic population: Facing the growing challenge. J. Natl. Med. Assoc..

[11] Peralta CA (2006). Risks for end-stage renal disease, cardiovascular events, and death in Hispanic versus non-Hispanic white adults with chronic kidney disease. J. Am. Soc. Nephrol..

[12] Desai N, Lora CM, Lash JP, Ricardo AC (2019). CKD and ESRD in US Hispanics. Am. J. Kidney Dis..

[13] Gordon EJ, Ladner DP, Caicedo JC, Franklin J (2010). Disparities in kidney transplant outcomes: A review. Semin. Nephrol..

[14] Anderson LM, Scrimshaw SC, Fullilove MT, Fielding JE, Normand J (2003). Culturally competent healthcare systems: A systematic review. Am. J. Prev. Med..

[15] Renzaho AMN, Romios P, Crock C, Sønderlund AL (2013). The effectiveness of cultural competence programs in ethnic minority patient-centered health care—A systematic review of the literature. Int. J. Qual. Health Care.

[16] Govere L, Govere EM (2016). How effective is cultural competence training of healthcare providers on improving patient satisfaction of minority groups? A systematic review of literature. Worldviews Evid. Based Nurs..

[17] Al Shamsi H, Almutairi AG, Al Mashrafi S, Al Kalbani T (2020). Implications of language barriers for healthcare: A systematic review. Oman Med. J..

[18] Fernandez A (2011). Language barriers, physician-patient language concordance, and glycemic control among insured Latinos with diabetes: The Diabetes Study of Northern California (DISTANCE). J. Gen. Intern. Med..

[19] Diamond L, Izquierdo K, Canfield D, Matsoukas K, Gany F (2019). A systematic review of the impact of patient-physician non-English language concordance on quality of care and outcomes. J. Gen. Intern. Med..

[20] Barwise AK, Curtis S, Diedrich DA, Pickering BW (2023). Using artificial intelligence to promote equitable care for inpatients with language barriers and complex medical needs: Clinical stakeholder perspectives. J. Am. Med. Inform. Assoc..

[21] Rosoł M, Gąsior JS, Łaba J, Korzeniewski K, Młyńczak M (2023). Evaluation of the performance of GPT-3.5 and GPT-4 on the Polish Medical Final Examination. Sci. Rep..

[22] Gan RK, Uddin H, Gan AZ, Yew YY, González PA (2023). ChatGPT’s performance before and after teaching in mass casualty incident triage. Sci. Rep..

[23] Bozza S (2023). A model-independent redundancy measure for human versus ChatGPT authorship discrimination using a Bayesian probabilistic approach. Sci. Rep..

[24] Walters WH, Wilder EI (2023). Fabrication and errors in the bibliographic citations generated by ChatGPT. Sci. Rep..

[25] Russe MF (2023). Performance of ChatGPT, human radiologists, and context-aware ChatGPT in identifying AO codes from radiology reports. Sci. Rep..

[26] Madrid-García A (2023). Harnessing ChatGPT and GPT-4 for evaluating the rheumatology questions of the Spanish access exam to specialized medical training. Sci. Rep..

[27] Miao J (2023). Performance of ChatGPT on nephrology test questions. Clin. J. Am. Soc. Nephrol..

[28] Garcia Valencia OA (2023). Enhancing kidney transplant care through the integration of chatbot. Healthcare (Basel).

[29] Herbold S, Hautli-Janisz A, Heuer U, Kikteva Z, Trautsch A (2023). A large-scale comparison of human-written versus ChatGPT-generated essays. Sci. Rep..

[30] Taloni A (2023). Comparative performance of humans versus GPT-4.0 and GPT-3.5 in the self-assessment program of American Academy of Ophthalmology. Sci. Rep..

[31] Nastasi AJ, Courtright KR, Halpern SD, Weissman GE (2023). A vignette-based evaluation of ChatGPT’s ability to provide appropriate and equitable medical advice across care contexts. Sci. Rep..

[32] Brin D (2023). Comparing ChatGPT and GPT-4 performance in USMLE soft skill assessments. Sci. Rep..

[33] Fütterer T (2023). ChatGPT in education: Global reactions to AI innovations. Sci. Rep..

[34] Jo H, Bang Y (2023). Analyzing ChatGPT adoption drivers with the TOEK framework. Sci. Rep..

[35] Breithaupt F (2024). Humans create more novelty than ChatGPT when asked to retell a story. Sci. Rep..

[36] Miao J, Thongprayoon C, Cheungpasitporn W (2024). Should artificial intelligence be used for physician documentation to reduce burnout?. Kidney.

[37] Frequently asked questions about kidney transplant evaluation and listing. https://optn.transplant.hrsa.gov/patients/by-organ/kidney/frequently-asked-questions-about-kidney-transplant-evaluation-and-listing/

[38] Kidney transplant FAQs. https://www.nhsbt.nhs.uk/organ-transplantation/kidney/is-a-kidney-transplant-right-for-you/kidney-transplant-faqs/

[39] Kidney Transplant. https://www.kidney.org/atoz/content/kidney-transplant

[40] OpenAI. Introducing ChatGPT. https://openai.com/blog/chatgpt (2023).

[41] Onder CE, Koc G, Gokbulut P, Taskaldiran I, Kuskonmaz SM (2024). Evaluation of the reliability and readability of ChatGPT-4 responses regarding hypothyroidism during pregnancy. Sci. Rep..

[42] Choi J (2024). Availability of ChatGPT to provide medical information for patients with kidney cancer. Sci. Rep..

[43] Peeters MJ, Sahloff EG, Stone GE (2010). A standardized rubric to evaluate student presentations. Am. J. Pharm. Educ..

[44] Pérez-Escamilla R, Garcia J, Song D (2010). Health care access among Hispanic immigrants:¿ Alguien está escuchando?[Is anybody listening?]. NAPA Bull..

[45] Caballero AE (2011). Understanding the Hispanic/Latino patient. Am. J. Med..

[46] Kaushik P, Reed B, Kalirai S, Perez-Nieves M (2020). Challenges in insulin initiation among Hispanics/Latinos with diabetes in the United States. Primary Care Diabetes.

[47] Brooks LA, Manias E, Bloomer MJ (2019). Culturally sensitive communication in healthcare: A concept analysis. Collegian.

[48] Shepherd SM, Willis-Esqueda C, Newton D, Sivasubramaniam D, Paradies Y (2019). The challenge of cultural competence in the workplace: Perspectives of healthcare providers. BMC Health Serv. Res..

[49] Handtke O, Schilgen B, Mösko M (2019). Culturally competent healthcare—A scoping review of strategies implemented in healthcare organizations and a model of culturally competent healthcare provision. PLoS One.

[50] Stap, D. & Araabi, A. in *Proceedings of the Workshop on Natural Language Processing for Indigenous Languages of the Americas* (*AmericasNLP*) 163–167.

[51] Costa-jussà, M. R. *et al.* No language left behind: Scaling human-centered machine translation. arXiv preprint arXiv:2207.04672 (2022).

[52] Manakhimova, S. *et al.* in *Proceedings of the Eighth Conference on Machine Translation* 224–245.

[53] Jiao, W., Wang, W., Huang, J., Wang, X. & Tu, Z. Is ChatGPT a good translator? Yes with GPT-4 as the engine. arXiv preprint arXiv:2301.08745 (2023).

[54] Siu, S. C. ChatGPT and GPT-4 for professional translators: Exploring the potential of large language models in translation. Available at SSRN 4448091 (2023).

