# Supplementary material for: AI-driven translations for kidney transplant equity in Hispanic populations
Source: Sci Rep. 2024 Apr 12;14:8511. doi: 10.1038/s41598-024-59237-7 (PMC11014982; doi:10.1038/s41598-024-59237-7)
Supplement: Supplementary file 1 — Supplementary Information. [file 41598_2024_59237_MOESM1_ESM.docx]

**Supplementary Table 1.** The score for linguistic accuracy and cultural sensitivity of GPT-3.5 and GPT-4.0 for individual FAQs

| **Chat GPT 3.5** | | | **Chat GPT 4.0** | | |
| --- | --- | --- | --- | --- | --- |
| **Question number** | **Accuracy** | **Culture** | **Question number** | **Accuracy** | **Culture** |
| **OPTN** | | | **OPTN** | | |
| 1 | 5 | 5 | 1 | 5 | 5 |
| 2 | 5 | 5 | 2 | 5 | 5 |
| 3 | 5 | 5 | 3 | 5 | 5 |
| 4 | 5 | 5 | 4 | 5 | 5 |
| 5 | 5 | 5 | 5 | 5 | 5 |
| 6 | 5 | 5 | 6 | 5 | 5 |
| 7 | 5 | 5 | 7 | 5 | 5 |
| 8 | 5 | 5 | 8 | 5 | 5 |
| 9 | 5 | 5 | 9 | 5 | 5 |
| 10 | 5 | 5 | 10 | 5 | 5 |
| 11 | 5 | 5 | 11 | 5 | 5 |
| 12 | 5 | 4 | 12 | 5 | 5 |
| 13 | 5 | 5 | 13 | 5 | 5 |
| 14 | 4 | 5 | 14 | 5 | 5 |
| 15 | 4 | 5 | 15 | 4 | 5 |
| 16 | 5 | 5 | 16 | 5 | 5 |
| 17 | 4 | 5 | 17 | 5 | 5 |
| 18 | 5 | 5 | 18 | 5 | 5 |
| 19 | 5 | 5 | 19 | 5 | 5 |
| **NHS** | | | **NHS** | | |
| 1 | 5 | 5 | 1 | 5 | 5 |
| 2 | 5 | 5 | 2 | 5 | 5 |
| 3 | 4 | 5 | 3 | 5 | 5 |
| 4 | 5 | 5 | 4 | 4 | 5 |
| 5 | 5 | 5 | 5 | 5 | 5 |
| 6 | 5 | 5 | 6 | 5 | 5 |
| 7 | 5 | 5 | 7 | 5 | 5 |
| 8 | 5 | 4 | 8 | 5 | 5 |
| 9 | 5 | 5 | 9 | 5 | 5 |
| 10 | 5 | 5 | 10 | 5 | 5 |
| 11 | 5 | 5 | 11 | 5 | 5 |
| 12 | 5 | 5 | 12 | 5 | 5 |
| 13 | 5 | 5 | 13 | 5 | 5 |
| 14 | 5 | 5 | 14 | 5 | 5 |
| 15 | 5 | 5 | 15 | 5 | 5 |
| **NKF** | | | **NKF** | | |
| 1 | 5 | 5 | 1 | 5 | 5 |
| 2 | 4 | 5 | 2 | 5 | 5 |
| 3 | 5 | 5 | 3 | 5 | 5 |
| 4 | 5 | 5 | 4 | 5 | 5 |
| 5 | 5 | 5 | 5 | 5 | 5 |
| 6 | 5 | 5 | 6 | 5 | 5 |
| 7 | 5 | 5 | 7 | 5 | 4 |
| 8 | 5 | 5 | 8 | 5 | 5 |
| 9 | 5 | 5 | 9 | 5 | 5 |
| 10 | 5 | 5 | 10 | 5 | 5 |
| 11 | 5 | 5 | 11 | 5 | 5 |
| 12 | 5 | 5 | 12 | 5 | 5 |
| 13 | 5 | 5 | 13 | 4 | 5 |
| 14 | 5 | 5 | 14 | 5 | 5 |
| 15 | 5 | 5 | 15 | 5 | 5 |
| 16 | 5 | 5 | 16 | 5 | 5 |
| 17 | 4 | 5 | 17 | 5 | 5 |
| 18 | 5 | 5 | 18 | 5 | 5 |
| 19 | 5 | 5 | 19 | 5 | 4 |
| 20 | 5 | 5 | 20 | 5 | 5 |

**Abbreviation**: NHS (National Health Service); NKF (National Kidney Foundation); OPTN (Organ Procurement and Transplantation Network)

RECIPIENT TRANSLATION SPANISH
CHAT GPT 3.5

**OPTN**


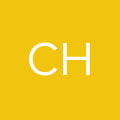


1. **You**

Please translate to Spanish: "How does a kidney transplant differ from dialysis? With dialysis treatment, a machine filters waste and extra fluid out of your blood the way your kidneys normally would. Many people undergo dialysis while awaiting a kidney transplant. Dialysis only filters the blood during a treatment session, not in the constant way a kidney does. For this reason, dialysis patients usually have diet and fluid restrictions. Dialysis also requires an access site – a vein or an implanted device where the blood can be collected and filtered outside the body. A kidney transplant is a surgical operation to give a functioning human kidney to someone whose kidneys have stopped working or are close to failing. The functioning kidney is removed from either a living donor or someone who has recently passed away. A transplanted kidney performs all the functions of a kidney a person has from birth. It constantly filters blood for waste and excess fluid. Most kidney transplant recipients do not have to limit their diet and fluid intake, but they do need to take medicine on a daily basis to help keep their transplant functioning."

ChatGPT

**ChatGPT**

"¿En qué se diferencia un trasplante de riñón de la diálisis? Con el tratamiento de diálisis, una máquina filtra los desechos y el exceso de líquido de la sangre de la misma manera que lo harían normalmente los riñones. Muchas personas se someten a la diálisis mientras esperan un trasplante de riñón.

La diálisis solo filtra la sangre durante una sesión de tratamiento, no de la manera constante como lo hace un riñón. Por esta razón, los pacientes en diálisis suelen tener restricciones en su dieta y en la ingesta de líquidos. La diálisis también requiere un sitio de acceso, ya sea una vena o un dispositivo implantado donde se pueda recoger la sangre y filtrarla fuera del cuerpo.

Un trasplante de riñón es una operación quirúrgica para proporcionar un riñón humano funcional a alguien cuyos riñones han dejado de funcionar o están cerca de fallar. El riñón funcional se extrae de un donante vivo o de alguien que ha fallecido recientemente.

Un riñón trasplantado realiza todas las funciones de un riñón que una persona tiene desde el nacimiento. Filtra constantemente la sangre en busca de desechos y líquido en exceso. La mayoría de los receptores de trasplante de riñón no tienen que limitar su dieta ni la ingesta de líquidos, pero sí deben tomar medicamentos a diario para ayudar a mantener funcionando el trasplante."


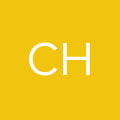


1. **You**

Please translate to Spanish: "What do I need to do to be considered for a transplant? To be considered for a kidney transplant, a transplant program must evaluate and accept you. You are not automatically listed for a transplant just because you begin dialysis or have some contact with a transplant program. Each transplant program makes its own decision about whether to accept someone for a transplant. The transplant team at each program has its own standards for accepting candidates. Each team may view the same facts and information different ways and make different decisions about listing a person for a transplant. Therefore, if one program is not willing to accept you as a candidate, a different program may accept you. Here are some common questions about what is involved with evaluation and testing, as well as factors that affect whether someone may be accepted for a transplant listing."

ChatGPT

**ChatGPT**

"¿Qué necesito hacer para ser considerado para un trasplante? Para ser considerado para un trasplante de riñón, un programa de trasplante debe evaluarte y aceptarte. No te incluyen automáticamente en la lista de espera para un trasplante solo porque comiences la diálisis o tengas algún contacto con un programa de trasplante.

Cada programa de trasplante toma su propia decisión sobre si aceptar a alguien para un trasplante. El equipo de trasplante de cada programa tiene sus propios estándares para aceptar candidatos. Cada equipo puede interpretar los mismos hechos e información de diferentes maneras y tomar decisiones distintas sobre la inclusión de una persona en la lista de espera para un trasplante. Por lo tanto, si un programa no está dispuesto a aceptarte como candidato, es posible que otro programa te acepte.

Aquí tienes algunas preguntas comunes sobre lo que implica la evaluación y las pruebas, así como los factores que afectan a la posibilidad de que alguien sea aceptado en la lista de espera para un trasplante."


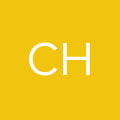


1. **You**

Please translate to Spanish: "Do I need a doctor’s referral, or can I contact the transplant program myself? You can contact the hospital yourself. You don’t need a doctor’s referral, but your doctor may have test results and medical history that will make it easier for the transplant team to evaluate you."

ChatGPT

**ChatGPT**

"¿Necesito una referencia médica o puedo ponerme en contacto con el programa de trasplante por mi cuenta? Puedes ponerte en contacto con el hospital por tu cuenta. No necesitas una referencia médica, pero tu médico puede tener resultados de pruebas y un historial médico que facilitarán la evaluación por parte del equipo de trasplante."


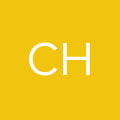


1. **You**

Please translate to Spanish: "Can I be evaluated for a transplant before I need dialysis? Yes. A standard test to assess your kidney function is your GFR (glomerular filtration rate). An ideal time to start the evaluation is when your GFR is between 25 and 30. Even if you haven’t reached end-stage kidney failure, starting the discussion early may give you time to consider your transplant options before you need dialysis."

ChatGPT

**ChatGPT**

"¿Puedo ser evaluado para un trasplante antes de necesitar diálisis? Sí. Una prueba estándar para evaluar la función de tus riñones es tu TFG (tasa de filtración glomerular). Un momento ideal para comenzar la evaluación es cuando tu TFG se encuentra entre 25 y 30. Incluso si no has llegado a la etapa final de insuficiencia renal, comenzar la discusión temprano puede darte tiempo para considerar tus opciones de trasplante antes de necesitar diálisis."


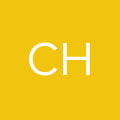


1. **You**

Please translate to Spanish: "What kinds of medical tests are done in an evaluation? The transplant team will do a number of medical tests. These include tests of your kidney function and other vital organs, screening for other diseases and medical conditions, and testing of your blood and genetic immune system markers to match you with compatible kidney offers. If you are on dialysis, the program also will want details about your dialysis treatment. If the program lists you for a transplant, it will also repeat some tests on a regular basis while you are on the waitlist."

ChatGPT

**ChatGPT**

"¿Qué tipos de pruebas médicas se realizan en una evaluación? El equipo de trasplante realizará una serie de pruebas médicas. Estas incluyen pruebas de la función de tus riñones y otros órganos vitales, detección de otras enfermedades y afecciones médicas, y pruebas de tu sangre y marcadores genéticos del sistema inmunológico para emparejarte con ofertas de riñones compatibles. Si estás en diálisis, el programa también querrá detalles sobre tu tratamiento de diálisis. Si el programa te incluye en la lista de espera para un trasplante, también repetirá algunas pruebas de manera regular mientras estés en la lista de espera."


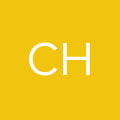


1. **You**

Please translate to Spanish: "How old is too old to be transplanted? There is no standard age limit to be transplanted. Each transplant program sets its own practice. For example, one program may not accept anyone older than 80 years of age; another may have no age limit. Most transplant candidates nationwide are age 50 or older, and many transplant programs often accept patients age 65 and older."

ChatGPT

**ChatGPT**

"¿Cuál es la edad máxima para ser trasplantado? No existe un límite de edad estándar para ser trasplantado. Cada programa de trasplante establece su propia práctica. Por ejemplo, un programa puede no aceptar a personas mayores de 80 años, mientras que otro puede no tener límite de edad. La mayoría de los candidatos a trasplantes en todo el país tienen 50 años o más, y muchos programas de trasplante suelen aceptar pacientes de 65 años o más."


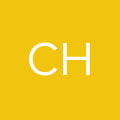


1. **You**

Please translate to Spanish: "What medical conditions might rule me out for a transplant listing? A few medical conditions might rule out a transplant. The transplant team will discuss these with you when you start your evaluation. In general, health issues that may keep you from having surgery or taking long-term medication may be reasons not to list you. These can include heart or lung conditions, current or recent cancer diagnosis, or morbid obesity. Depending on the medical issues involved, the program may have another specialist examine you and advise whether transplantation would be a safe option. In some cases, even if a transplant program doesn’t accept you immediately for a transplant, it may reconsider you later if your condition improves (for example, you lose weight as directed, or your cancer goes into remission). You also might consider applying to a different transplant center, since each hospital has its own criteria for accepting transplant candidates."

ChatGPT

**ChatGPT**

"¿Qué condiciones médicas podrían descartarme para ser incluido en la lista de trasplantes? Algunas condiciones médicas podrían descartarte para un trasplante. El equipo de trasplante discutirá estas cuestiones contigo cuando comiences tu evaluación.

En general, problemas de salud que puedan impedirte someterte a una cirugía o tomar medicación a largo plazo pueden ser razones para no incluirte en la lista. Estos pueden incluir condiciones cardíacas o pulmonares, un diagnóstico actual o reciente de cáncer u obesidad mórbida. Dependiendo de los problemas médicos involucrados, el programa puede hacer que otro especialista te examine y te aconseje si el trasplante sería una opción segura.

En algunos casos, incluso si un programa de trasplante no te acepta de inmediato para un trasplante, puede reconsiderarte más adelante si tu condición mejora (por ejemplo, si pierdes peso según lo indicado o si tu cáncer entra en remisión). También podrías considerar solicitar en otro centro de trasplantes, ya que cada hospital tiene sus propios criterios para aceptar candidatos a trasplante."


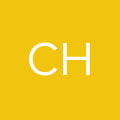


1. **You**

Please translate to Spanish: "Does a history of drug or alcohol abuse affect a transplant decision? A past history of drug or alcohol abuse may not rule out a person who has ended use and/or shown progress in treatment. During your evaluation, a social worker will meet with you to help the transplant team assess how your current behavior may affect your ability to do well with a transplant in the long term."

ChatGPT

**ChatGPT**

"¿Afecta el historial de abuso de drogas o alcohol a la decisión de un trasplante? Un historial pasado de abuso de drogas o alcohol puede no descartar a una persona que haya dejado de usar y/o haya mostrado avances en el tratamiento. Durante tu evaluación, un trabajador social se reunirá contigo para ayudar al equipo de trasplante a evaluar cómo tu comportamiento actual puede afectar tu capacidad para tener éxito a largo plazo con un trasplante."


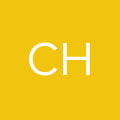


1. **You**

Please translate to Spanish: "Do I need medical insurance at the time I get evaluated? It’s important for the long-term success of your transplant for you to have insurance or other financial resources to cover needed expenses. Even after the transplant operation, you will take anti-rejection drugs and need follow-up medical care as long as you have a transplant. Every transplant program has a social worker or financial coordinator who can advise you on insurance or funding options."

ChatGPT

**ChatGPT**

"¿Necesito contar con un seguro médico en el momento de ser evaluado? Es importante para el éxito a largo plazo de tu trasplante que cuentes con un seguro médico u otros recursos financieros para cubrir los gastos necesarios. Incluso después de la operación de trasplante, deberás tomar medicamentos antirrechazo y necesitarás atención médica de seguimiento mientras tengas un trasplante. Cada programa de trasplante tiene un trabajador social o coordinador financiero que puede asesorarte sobre opciones de seguro o financiamiento."


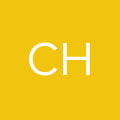


1. **You**

Please translate to Spanish: "Do I need to have a stable housing situation to be evaluated or listed for a transplant? It is best if you have a stable housing situation, so the transplant team can contact you for appointments and any potential organ offer after you are listed for transplant. A social worker with the transplant program may be able to help you if you don’t have a permanent place to stay."

ChatGPT

**ChatGPT**

"¿Necesito tener una situación de vivienda estable para ser evaluado o incluido en la lista de trasplantes? Es preferible que tengas una situación de vivienda estable, de manera que el equipo de trasplante pueda ponerse en contacto contigo para citas y cualquier posible oferta de órganos una vez que estés en la lista de trasplante. Un trabajador social del programa de trasplante puede ayudarte si no tienes un lugar permanente donde quedarte."


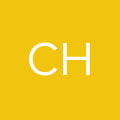


1. **You**

Please translate to Spanish: "What else will the transplant team want to know about me? You will talk with a psychologist and/or a social worker, who are members of the transplant team. The team will want to be sure you can understand and follow medical instructions. They also will want to know what social support you have (family, friends and/or caregivers) to help you with tasks such as attending appointments and taking your medications. This is important to help ensure you will do well long after the transplant operation. If you are accepted for a transplant, the transplant team will need emergency contact information for you and for any backup person you name. This helps them contact you quickly for matching kidney offers. You also will need to set and keep appointments on a regular basis to allow the transplant staff to check your condition while you are waiting for a transplant."

ChatGPT

**ChatGPT**

"¿Qué más querrá saber el equipo de trasplante sobre mí? Hablarás con un psicólogo y/o un trabajador social, quienes son miembros del equipo de trasplante. El equipo querrá asegurarse de que puedas comprender y seguir las instrucciones médicas. También querrán saber qué apoyo social tienes (familia, amigos y/o cuidadores) para ayudarte con tareas como asistir a citas y tomar tus medicamentos. Esto es importante para asegurarse de que te irá bien mucho tiempo después de la operación de trasplante.

Si eres aceptado para un trasplante, el equipo de trasplante necesitará información de contacto de emergencia para ti y para cualquier persona de respaldo que nombres. Esto les ayuda a contactarte rápidamente en caso de ofertas de riñón compatibles. También deberás programar y asistir a citas de forma regular para permitir que el personal de trasplante revise tu condición mientras esperas un trasplante."


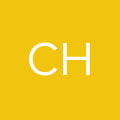


1. **You**

User Please translate to Spanish: "Can I list at more than one transplant program at the same time? It is possible to list at multiple transplant programs, but each program would decide on its own whether to accept and list you. You would need to meet all the requirements the transplant team sets at any program where you are listed. Transplant candidates listed at a program close to the organ donor hospital are usually considered for matching offers before those at more distant hospitals. Listing at programs in different areas of the country might help someone find a match more quickly."

ChatGPT

**ChatGPT**

"¿Puedo inscribirme en más de un programa de trasplante al mismo tiempo? Es posible inscribirse en varios programas de trasplante, pero cada programa decidiría por sí mismo si te acepta y te incluye en su lista. Deberías cumplir con todos los requisitos establecidos por el equipo de trasplante en cualquier programa en el que estés inscrito.

Por lo general, los candidatos a trasplante inscritos en un programa cercano al hospital donante de órganos suelen ser considerados para ofertas de compatibilidad antes que los que están en hospitales más distantes. Inscribirse en programas en diferentes áreas del país podría ayudar a alguien a encontrar una coincidencia más rápidamente."


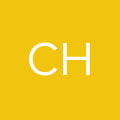


1. **You**

Please translate to Spanish: "How does the transplant process work? Kidneys can be transplanted from living or deceased donors. Below are common questions relating to living donor transplants."

ChatGPT

**ChatGPT**

"¿Cómo funciona el proceso de trasplante? Los riñones pueden ser trasplantados de donantes vivos o fallecidos. A continuación, se presentan preguntas comunes relacionadas con los trasplantes de donantes vivos."


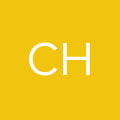


1. **You**

Please translate to Spanish: "Who can be a living donor? A living donor may be a family member, friend, coworker, neighbor, or even a complete stranger, as long as they are a medical match for you. Your transplant team can advise you how to discuss living donation with people who might be interested. All living donors must make a free and independent choice. They must understand there are medical risks to donating a kidney. Life expectancy - Donating a kidney is major surgery but the procedure has not been shown to reduce the donor’s life expectancy. Surgical Complications – Donors face possible post-operative complications such as bleeding, wound infection, fever, etc., but most complications are generally short- term and can be treated with quality medical care. Recovery Time - Recovery after surgery should take between three and six weeks. You may need to stay in the hospital for up to seven days. Your doctor or surgeon will discuss the success of the surgery and any follow-up treatments you may need. Living donors will have a number of medical tests to help ensure they are healthy enough to donate. They also will talk to a psychologist and/or social worker to help the transplant team make sure they have realistic expectations and reasons for donation."

ChatGPT

**ChatGPT**

"¿Quién puede ser un donante vivo? Un donante vivo puede ser un miembro de la familia, un amigo, un compañero de trabajo, un vecino o incluso un desconocido, siempre y cuando sean una coincidencia médica contigo. Tu equipo de trasplante puede aconsejarte sobre cómo hablar con personas que puedan estar interesadas en la donación de órganos.

Todos los donantes vivos deben tomar una decisión libre e independiente. Deben comprender que existen riesgos médicos asociados con la donación de un riñón.

Expectativa de vida - Donar un riñón es una cirugía importante, pero no se ha demostrado que la intervención reduzca la expectativa de vida del donante. Complicaciones quirúrgicas - Los donantes pueden enfrentar posibles complicaciones postoperatorias como sangrado, infección de la herida, fiebre, etc., pero la mayoría de las complicaciones suelen ser de corto plazo y pueden tratarse con atención médica de calidad. Tiempo de recuperación - La recuperación después de la cirugía debería llevar entre tres y seis semanas. Es posible que debas permanecer en el hospital hasta siete días. Tu médico o cirujano discutirá el éxito de la cirugía y cualquier tratamiento de seguimiento que puedas necesitar. Los donantes vivos se someterán a una serie de pruebas médicas para garantizar que estén lo suficientemente sanos como para donar. También hablarán con un psicólogo y/o trabajador social para ayudar al equipo de trasplante a asegurarse de que tengan expectativas realistas y razones válidas para la donación."


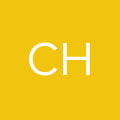


1. **You**

Please translate to Spanish: "What is kidney paired donation? If you have a potential donor who is not a biologic match with you (such as having an incompatible blood type), but may match with someone else, kidney paired donation may be an option. In kidney paired donation, information about potential living donors and recipients is matched in a computer to find successful donor-recipient combinations. So the person who wanted to donate to you can donate to a different patient, and that patient’s intended donor can donate a kidney to you. Kidney paired donation may often help transplant candidates who are very hard to match."

ChatGPT

**ChatGPT**

"¿Qué es la donación cruzada de riñón? Si tienes un posible donante que no es una coincidencia biológica contigo (como tener un tipo de sangre incompatible), pero que puede coincidir con otra persona, la donación cruzada de riñón puede ser una opción.

En la donación cruzada de riñón, la información sobre posibles donantes vivos y receptores se empareja en una computadora para encontrar combinaciones exitosas de donantes y receptores. Entonces, la persona que quería donar a ti puede donar a otro paciente, y el donante previsto de ese paciente puede donarte un riñón a ti. La donación cruzada de riñón a menudo puede ayudar a los candidatos a trasplante que son muy difíciles de emparejar."


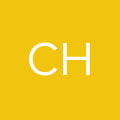


1. **You**

Please translate to Spanish: "How long does it take for a living donor transplant? That depends on a number of factors, but a wait of several weeks to a few months is common. Living donor transplants are routinely scheduled operations. The donation and transplant procedures are closely timed so that the transplant has the best chance of success."

ChatGPT

**ChatGPT**

"¿Cuánto tiempo lleva un trasplante de donante vivo? Eso depende de varios factores, pero una espera de varias semanas a unos pocos meses es común. Los trasplantes de donantes vivos son operaciones programadas de manera rutinaria. Los procedimientos de donación y trasplante se programan de manera cercana para que el trasplante tenga la mejor oportunidad de éxito."


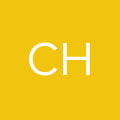


1. **You**

Please translate to Spanish: "How would I be matched for a kidney from a deceased donor? The computer database of the Organ Procurement and Transplantation Network (OPTN) matches medical information on all transplant candidates who are actively listed on the waitlist nationwide with medical information on deceased donors. The database produces a rank-ordered list for each donor offer that determines who will be offered the kidney. When a transplant team gets an organ offer for one of its patients, it reviews detailed medical information about the donor. The team will then decide whether to accept the offer for their candidate. If the offer is turned down for one candidate, it goes to the next person on the list. Kidneys that are likely to function for the longest amount of time are considered first for candidates who are likely to need them the longest. Other factors in matching include how closely the donor’s blood and tissue type matches the patient, how long the candidate has needed a kidney transplant, and how close the transplant hospital is to the donor hospital."

ChatGPT

**ChatGPT**

"¿Cómo sería mi compatibilidad con un riñón de un donante fallecido? La base de datos informática de la Red de Obtención y Trasplante de Órganos (OPTN, por sus siglas en inglés) compara la información médica de todos los candidatos a trasplante que están activamente inscritos en la lista de espera a nivel nacional con la información médica de los donantes fallecidos. La base de datos genera una lista ordenada por prioridad para cada oferta de donante que determina quién recibirá el riñón.

Cuando un equipo de trasplante recibe una oferta de órgano para uno de sus pacientes, revisa información médica detallada sobre el donante. El equipo luego decide si aceptar la oferta para su candidato. Si la oferta es rechazada para un candidato, pasa a la siguiente persona en la lista.

Los riñones que es más probable que funcionen durante el mayor período de tiempo se consideran primero para los candidatos que es más probable que los necesiten durante más tiempo. Otros factores en la compatibilidad incluyen cuán cercano es el tipo de sangre y tejido del donante al del paciente, cuánto tiempo ha necesitado el candidato un trasplante de riñón y cuán cerca está el hospital de trasplantes del hospital del donante."


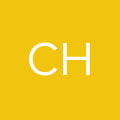


1. **You**

Please translate to Spanish: "How long is the wait for a deceased donor transplant? Unfortunately, there are not enough organs available for everyone who needs a kidney transplant. Waiting times for a deceased donor kidney transplant can vary greatly in different parts of the country, but waiting times of 4 to 5 years are common. Some people may wait much shorter or longer based on how well they match with most donors. Your transplant program can tell you more about how long you might expect to wait."

ChatGPT

**ChatGPT**

"¿Cuánto dura la espera para un trasplante de donante fallecido? Lamentablemente, no hay suficientes órganos disponibles para todos los que necesitan un trasplante de riñón. Los tiempos de espera para un trasplante de riñón de donante fallecido pueden variar ampliamente en diferentes partes del país, pero es común esperar de 4 a 5 años. Algunas personas pueden esperar mucho menos o mucho más tiempo según cuánto se asemejen a la mayoría de los donantes. Tu programa de trasplante puede proporcionarte más información sobre cuánto tiempo puedes esperar."


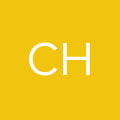


1. **You**

Please translate to Spanish: "What must I do after a transplant? A transplant is a treatment for kidney failure, not a cure. After a transplant, you will need ongoing treatment to stay healthy and keep your kidney working well. Your immune system will recognize the transplanted kidney as being “different.” It may try to attack (reject) the organ. To lower the chance of organ rejection, you will need to take medicine every day. Your transplant team wants to make sure your kidney is functioning properly and that you’re taking the right doses of medicine to prevent or treat organ rejection. As long as you have a functioning transplant, you will need to have regular medical tests and appointments. These tests and appointments are more frequent just after your transplant. If you do well in the longer term, you do not need to have them as often."

ChatGPT

**ChatGPT**

"¿Qué debo hacer después de un trasplante? Un trasplante es un tratamiento para la insuficiencia renal, no una cura. Después de un trasplante, necesitarás tratamiento continuo para mantenerte saludable y mantener funcionando bien tu riñón.

Tu sistema inmunológico reconocerá el riñón trasplantado como algo "diferente". Puede intentar atacar (rechazar) el órgano. Para reducir la probabilidad de rechazo del órgano, deberás tomar medicamentos todos los días. Tu equipo de trasplante quiere asegurarse de que tu riñón funcione adecuadamente y de que estés tomando las dosis correctas de medicamentos para prevenir o tratar el rechazo del órgano.

Mientras tengas un trasplante funcionando, deberás someterte a pruebas médicas y citas regulares. Estas pruebas y citas son más frecuentes justo después de tu trasplante. Si te va bien a largo plazo, no necesitarás hacerlas tan seguido."

**NHS**


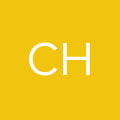


1. **You**

Please translate to Spanish: "What is the life expectancy of a kidney transplant patient? Life expectancy for patients with kidney disease is very variable depending on age and other medical issues. Please discuss this with the team caring for you."

ChatGPT

**ChatGPT**

"¿Cuál es la esperanza de vida de un paciente con trasplante de riñón? La esperanza de vida de los pacientes con enfermedad renal es muy variable y depende de la edad y otros problemas médicos. Por favor, discuta esto con el equipo que lo está atendiendo."


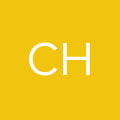


1. **You**

Please translate to Spanish: "What is the life expectancy of a transplanted kidney? On average, a kidney transplant from a living donor lasts for 20-25 years, while a kidney from a deceased donor lasts 15-20 years. This is very variable depending on the age of the donor and patient, and other medical issues. Please speak to your kidney care team for more personalised information. Some patients are able to have additional kidney transplants after this."

ChatGPT

**ChatGPT**

"¿Cuál es la esperanza de vida de un riñón trasplantado? En promedio, un riñón trasplantado de un donante vivo dura de 20 a 25 años, mientras que un riñón de un donante fallecido dura de 15 a 20 años.

Esto es muy variable y depende de la edad del donante y del paciente, así como de otros problemas médicos. Por favor, hable con su equipo de cuidado renal para obtener información más personalizada.

Algunos pacientes pueden someterse a trasplantes de riñón adicionales después de esto."


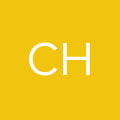


1. **You**

Please translate to Spanish: "How successful are kidney transplants in UK? For patients that receive a living donor kidney transplant, 99 out of 100 patients are alive after one year and 86 out of 100 are alive after ten years. For patients that receive a deceased donor kidney transplant, 96 to 97 out of 100 patients are alive after one year and 72 to 76 out of 100 are alive after ten years."

ChatGPT

**ChatGPT**

"¿Qué tan exitosos son los trasplantes de riñón en el Reino Unido? Para los pacientes que reciben un trasplante de riñón de un donante vivo, 99 de cada 100 pacientes están vivos después de un año y 86 de cada 100 están vivos después de diez años.

Para los pacientes que reciben un trasplante de riñón de un donante fallecido, entre el 96 y el 97 de cada 100 pacientes están vivos después de un año y entre el 72 y el 76 de cada 100 están vivos después de diez años."


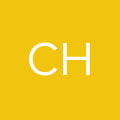


1. **You**

Please translate to Spanish: "What is the longest a transplanted kidney has lasted? Some patients have had a kidney transplant that has kept working for more than 40 years. Up-to-date records on the longest functioning kidney transplant are not kept."

ChatGPT

**ChatGPT**

"¿Cuál es el tiempo más largo que ha durado un riñón trasplantado? Algunos pacientes han tenido un trasplante de riñón que ha seguido funcionando durante más de 40 años. No se llevan registros actualizados sobre el trasplante de riñón que haya tenido la duración más larga."


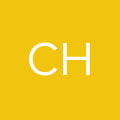


1. **You**

Please translate to Spanish: "What is the maximum age for a kidney transplant? All decisions on transplantation are made on a case-by-case basis. If you have concerns that you might be too old for a kidney transplant, please discuss this with your kidney care team."

ChatGPT

**ChatGPT**

"¿Cuál es la edad máxima para un trasplante de riñón? Todas las decisiones sobre el trasplante se toman caso por caso. Si tiene preocupaciones de que pueda ser demasiado mayor para un trasplante de riñón, por favor, discútalo con su equipo de atención renal."


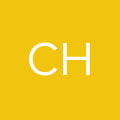


1. **You**

Please translate to Spanish: "How long is the wait for a kidney? The average wait for a deceased donor kidney transplant in the UK is 2-3 years. If you have a living donor, it usually takes just 3-6 months to have the required tests on you and your donor and to arrange the transplant."

ChatGPT

**ChatGPT**

"¿Cuánto tiempo es la espera para un riñón? La espera promedio para un trasplante de riñón de donante fallecido en el Reino Unido es de 2-3 años.

Si tiene un donante vivo, generalmente se necesitan solo de 3 a 6 meses para realizar las pruebas requeridas tanto en usted como en su donante y para organizar el trasplante."


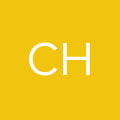


1. **You**

Please translate to Spanish: "How long will I need to be in hospital after a kidney transplant? If you’re receiving a kidney from a living donor – the usual stay is 4 to 6 days. If you’re receiving a kidney from a deceased donor - most patients stay in hospital for 6 to 10 days."

ChatGPT

**ChatGPT**

"¿Cuánto tiempo necesitaré estar en el hospital después de un trasplante de riñón? Si está recibiendo un riñón de un donante vivo, la estadía habitual es de 4 a 6 días.

Si está recibiendo un riñón de un donante fallecido, la mayoría de los pacientes permanecen en el hospital de 6 a 10 días."


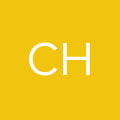


1. **You**

Please translate to Spanish: "How long does it take to recover from a kidney transplant? Everyone recovers at a different rate after a kidney transplant. For patients that worked before the transplant, they are usually back to work within 2-3 months of the transplant. Once the first few months after your operation have passed, it is likely that your kidney function will be stable and you won’t need to come to the transplant clinic as often. After the first 3-6 months, the risk of transplant rejection is lower and your medicines will usually reduce."

ChatGPT

**ChatGPT**

"¿Cuánto tiempo se necesita para recuperarse de un trasplante de riñón? Todos se recuperan a diferentes ritmos después de un trasplante de riñón. Para los pacientes que trabajaban antes del trasplante, generalmente vuelven al trabajo en un plazo de 2 a 3 meses después del trasplante. Una vez que hayan pasado los primeros meses después de la operación, es probable que su función renal sea estable y que no necesite venir a la clínica de trasplantes con tanta frecuencia. Después de los primeros 3-6 meses, el riesgo de rechazo del trasplante es menor y, por lo general, sus medicamentos se reducirán."


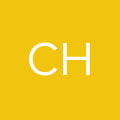


1. **You**

Please translate to Spanish: "Can I die from a kidney transplant? As with any major operation, there is a small risk of dying, but this is rare. The average risk of dying during the first few months after a kidney transplant is between 1-2 in 100 patients. However, this varies widely on a patient-by-patient basis."

ChatGPT

**ChatGPT**

"¿Puedo morir por un trasplante de riñón? Como con cualquier operación importante, existe un pequeño riesgo de morir, pero esto es raro. El riesgo promedio de morir durante los primeros meses después de un trasplante de riñón es de entre 1-2 de cada 100 pacientes. Sin embargo, esto varía ampliamente de un paciente a otro."


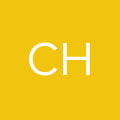


1. **You**

Please translate to Spanish: "Will my original kidney disease come back after the transplant? This can happen with some patients and is known as ‘recurrence’. Some kidney diseases can come back and affect the kidney transplant very quickly (days to weeks), but this is very rare. If this is a possibility, your kidney doctors and transplant surgeons will discuss this with you. If you have had difficult medical problems before you developed kidney failure, this may make it more difficult to have a transplant. You will have a number of different medical tests to see if a kidney transplant is the best treatment option for you."

ChatGPT

**ChatGPT**

"¿Mi enfermedad renal original volverá después del trasplante? Esto puede suceder en algunos pacientes y se conoce como 'recurrencia'.

Algunas enfermedades renales pueden volver y afectar al trasplante de riñón muy rápidamente (en días o semanas), pero esto es muy raro. Si esta es una posibilidad, sus médicos especialistas en riñones y cirujanos de trasplantes lo discutirán con usted. Si ha tenido problemas médicos difíciles antes de desarrollar la insuficiencia renal, esto puede dificultar la realización de un trasplante. Se le realizarán diversas pruebas médicas para determinar si un trasplante de riñón es la mejor opción de tratamiento para usted."


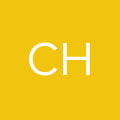


1. **You**

Please translate to Spanish: "Can I have another kidney transplant if my transplant stops working? Yes. It is possible to have multiple kidney transplants. You will need to either receive a kidney from a living donor or go back on the national transplant waiting list."

ChatGPT

**ChatGPT**

"¿Puedo tener otro trasplante de riñón si mi trasplante deja de funcionar? Sí. Es posible tener múltiples trasplantes de riñón. Necesitará recibir un riñón de un donante vivo o volver a inscribirse en la lista nacional de espera para trasplantes."


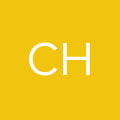


1. **You**

Please translate to Spanish: "Can I donate an organ if I’ve had a transplant? Yes, this is possible. Your general health will be taken into account. Please consider recording your wishes on organ donation in the national register."

ChatGPT

**ChatGPT**

"¿Puedo donar un órgano si he tenido un trasplante? Sí, esto es posible. Se tendrá en cuenta su salud general. Por favor, considere registrar sus deseos sobre la donación de órganos en el registro nacional."


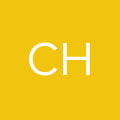


1. **You**

Please translate to Spanish: "There were 2,868 adult kidney transplants performed in 2021/2022 in the UK."

ChatGPT

**ChatGPT**

"Se realizaron 2,868 trasplantes de riñón en adultos en el Reino Unido en 2021/2022."


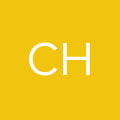


1. **You**

Please translate to Spanish: "How many people are waiting for kidney transplants in the UK? In the UK, around 5,000 people are in need of a kidney transplant."

ChatGPT

**ChatGPT**

"¿Cuántas personas están esperando un trasplante de riñón en el Reino Unido? En el Reino Unido, alrededor de 5,000 personas necesitan un trasplante de riñón."


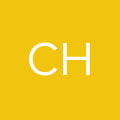


1. **You**

Please translate to Spanish: "How long is kidney transplant surgery? Kidney transplant surgery takes between 2-4 hours."

ChatGPT

**ChatGPT**

"¿Cuánto tiempo dura la cirugía de trasplante de riñón? La cirugía de trasplante de riñón dura entre 2 y 4 horas."

**NKF**


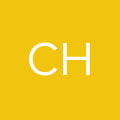


1. **You**

Please translate to Spanish: "What is a kidney transplant? When you get a kidney transplant, a healthy kidney is placed inside your body to do the work your own kidneys can no longer do. The healthy kidney can come from someone has died and chosen to donate, called a deceased donor, or from someone who has two healthy kidneys and chooses to donate one, called a living donor. A successful kidney transplant may allow you to live longer and to live the kind of life you were living before you got kidney disease. For many patients, there are fewer limits on what you can eat and drink, though you should follow a heart-healthy diet and maintain a healthy weight to help your new kidney last. Your health and energy should also improve. Studies show that people with kidney transplants live longer than those who remain on dialysis. Having a kidney transplant does not “cure” kidney disease. There are also risks, including the risks of surgery. After the transplant, you will need to take anti-rejection medicines for as long as your new kidney is working, which can have side effects. You will have a higher risk for infections and certain types of cancer. Although most transplants are successful and last for many years, how long they last can vary from one person to the next. Depending on your age, many people will need more than one kidney transplant during a lifetime."

ChatGPT

**ChatGPT**

"¿Qué es un trasplante de riñón?

Cuando te sometes a un trasplante de riñón, se coloca un riñón sano dentro de tu cuerpo para realizar el trabajo que tus propios riñones ya no pueden hacer. El riñón sano puede provenir de alguien que ha fallecido y eligió donar, llamado donante fallecido, o de alguien que tiene dos riñones sanos y elige donar uno, llamado donante vivo.

Un trasplante de riñón exitoso puede permitirte vivir más tiempo y llevar el tipo de vida que tenías antes de contraer una enfermedad renal. Para muchos pacientes, hay menos restricciones en lo que puedes comer y beber, aunque debes seguir una dieta saludable para el corazón y mantener un peso saludable para ayudar a que tu nuevo riñón perdure. Tu salud y energía también deberían mejorar. Los estudios muestran que las personas con trasplantes de riñón viven más tiempo que aquellos que permanecen en diálisis.

Tener un trasplante de riñón no "cura" la enfermedad renal. También existen riesgos, incluyendo los riesgos de la cirugía. Después del trasplante, necesitarás tomar medicamentos para prevenir el rechazo durante el tiempo que tu nuevo riñón esté funcionando, lo que puede tener efectos secundarios. Tendrás un mayor riesgo de infecciones y ciertos tipos de cáncer.

Aunque la mayoría de los trasplantes son exitosos y duran muchos años, la duración puede variar de una persona a otra. Dependiendo de tu edad, es probable que muchas personas necesiten más de un trasplante de riñón durante toda su vida."


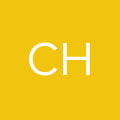


1. **You**

Please translate to Spanish: "What is a “preemptive” or “early” transplant? Getting a transplant before you need to start dialysis is called a preemptive transplant. It allows you to avoid dialysis altogether. Getting a transplant not long after kidneys fail (but with some time on dialysis) is referred to as an early transplant. Both have benefits. Some research shows that a pre-emptive or early transplant, with little or no time spent on dialysis, can lead to better long-term health. It may also allow you to keep working, save time and money, and have a better quality of life. If your doctor has told you that your kidney disease is progressing or that you are in a later stage of kidney disease, called Stage 3B, Stage 4, or Stage 5, it could be a good time to talk about kidney transplant."

ChatGPT

**ChatGPT**

"¿Qué es un trasplante "preventivo" o "temprano"?

Obtener un trasplante antes de que necesites comenzar la diálisis se llama un trasplante preventivo. Te permite evitar la diálisis por completo. Obtener un trasplante poco después de que los riñones fallen (pero con cierto tiempo en diálisis) se denomina trasplante temprano. Ambos tienen beneficios. Algunas investigaciones muestran que un trasplante preventivo o temprano, con poco o ningún tiempo en diálisis, puede llevar a una mejor salud a largo plazo. También puede permitirte seguir trabajando, ahorrar tiempo y dinero, y tener una mejor calidad de vida. Si tu médico te ha dicho que tu enfermedad renal está progresando o que te encuentras en una etapa avanzada de la enfermedad renal, llamada Etapa 3B, Etapa 4 o Etapa 5, podría ser un buen momento para hablar sobre el trasplante de riñón."


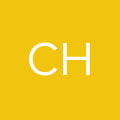


1. **You**

Please translate to Spanish: "Who can get a kidney transplant? Kidney patients of all ages—from children to seniors—are able to consider a transplant. For most people, getting a transplant can be a good treatment choice. Every person being considered for transplant will complete a full medical and psychosocial evaluation at a transplant center to make sure they are a good candidate. The evaluation helps find any problems, so they can be corrected before transplant. You must be healthy enough to have the operation. You must also be free from cancer and infection."

ChatGPT

**ChatGPT**

"¿Quiénes pueden recibir un trasplante de riñón?

Los pacientes de riñón de todas las edades, desde niños hasta personas mayores, pueden considerar un trasplante. Para la mayoría de las personas, recibir un trasplante puede ser una buena opción de tratamiento.

Toda persona que esté siendo considerada para un trasplante completará una evaluación médica y psicosocial completa en un centro de trasplantes para asegurarse de que sean un buen candidato. La evaluación ayuda a detectar cualquier problema, para que puedan ser corregidos antes del trasplante. Debes estar lo suficientemente sano como para someterte a la operación. También debes estar libre de cáncer e infecciones."


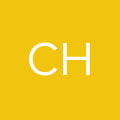


1. **You**

Please translate to Spanish: "What if I’m older or have other health problems? In many cases, people who are older or have other health conditions like diabetes can still have successful kidney transplants. Careful evaluation at a transplant center is needed to understand and deal with any special risks. You may be asked to do some things that can lessen certain risks and improve the chances of a successful transplant. For example, you may be asked to lose weight or quit smoking. Only a transplant center can decide if you are healthy enough to receive a kidney transplant. If you have diabetes, you may also be able to have a pancreas transplant. Ask your healthcare professional about getting a pancreas transplant along with a kidney transplant."

ChatGPT

**ChatGPT**

"¿Qué sucede si soy mayor o tengo otros problemas de salud?

En muchos casos, las personas que son mayores o tienen otras condiciones de salud como la diabetes aún pueden tener trasplantes de riñón exitosos. Se necesita una evaluación cuidadosa en un centro de trasplantes para comprender y abordar cualquier riesgo especial. Es posible que se te pida que hagas algunas cosas que pueden reducir ciertos riesgos y mejorar las posibilidades de un trasplante exitoso. Por ejemplo, es posible que se te pida que pierdas peso o que dejes de fumar. Solo un centro de trasplantes puede decidir si estás lo suficientemente sano como para recibir un trasplante de riñón.

Si tienes diabetes, es posible que también puedas recibir un trasplante de páncreas. Consulta a tu profesional de la salud acerca de la posibilidad de recibir un trasplante de páncreas junto con un trasplante de riñón."


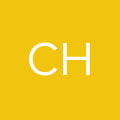


1. **You**

Please translate to Spanish: "How will I pay for a transplant? Medicare covers about 80% of the costs associated with an evaluation, transplant operation, follow-up care, and anti-rejection medicines. Private insurance through your job and state insurance programs may cover some costs as well. However, your post-transplant expenses may only be covered for a limited number of years. It’s important to discuss insurance coverage with your social worker and the transplant financial coordinator, who can answer your questions or direct you to others who can help. "

ChatGPT

**ChatGPT**

"¿Cómo pagaré un trasplante?

Medicare cubre aproximadamente el 80% de los costos asociados con una evaluación, la operación de trasplante, la atención de seguimiento y los medicamentos antirrechazo. El seguro privado a través de tu trabajo y los programas de seguro estatal también pueden cubrir algunos costos. Sin embargo, es posible que tus gastos posteriores al trasplante solo estén cubiertos durante un número limitado de años. Es importante discutir la cobertura del seguro con tu trabajador social y el coordinador financiero del trasplante, quienes pueden responder a tus preguntas o dirigirte a otras personas que puedan ayudarte."


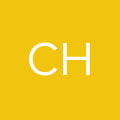


1. **You**

Please translate to Spanish: "How does the evaluation process work? The evaluation process for a transplant is very thorough. Your healthcare team will need to know a lot about you to help them—and you—decide if a transplant is right for you. Medical professionals will give you a complete physical exam, review your health records, and order a series of tests and X-rays to learn about your overall health. Everything that can affect how well you can handle a transplant will be checked. One thing you can do to speed the process is to get all the testing done as quickly as possible and stay in close contact with the transplant team. If you’re told you might not be right for a transplant, don’t be afraid to ask why. You can also consider doing an evaluation at another center if one center says no. Remember, being active in your own care is one of the best ways to stay healthy. If someone you know would like to donate a kidney to you, that person will also need to go through a screening evaluation to find out if he or she is healthy enough to donate. If it’s your child who has kidney disease, transplant can be the best treatment for them. Transplantation allows children and young adults to develop in as normal a way as possible in their formative years. If your child has kidney disease, you’ll want to give serious thought to getting a transplant evaluation for him or her. If the evaluation process shows that a transplant is right for you or your child, the next step is getting a suitable kidney. (See "Finding a Kidney" below.)"

ChatGPT

**ChatGPT**

"¿Cómo funciona el proceso de evaluación? El proceso de evaluación para un trasplante es muy exhaustivo. Tu equipo de atención médica necesitará conocer muchos detalles sobre ti para ayudarlos a ti y a ellos a decidir si un trasplante es adecuado para ti. Los profesionales médicos te realizarán un examen físico completo, revisarán tus registros de salud y ordenarán una serie de pruebas y radiografías para conocer tu estado de salud en general. Se revisará todo lo que pueda afectar la forma en que podrías manejar un trasplante.

Una cosa que puedes hacer para acelerar el proceso es completar todas las pruebas lo más rápido posible y mantener un contacto cercano con el equipo de trasplante. Si te dicen que es posible que no seas adecuado para un trasplante, no dudes en preguntar por qué. También puedes considerar hacer una evaluación en otro centro si uno te dice que no. Recuerda que ser activo en tu propio cuidado es una de las mejores formas de mantenerse saludable.

Si alguien que conoces quiere donarte un riñón, esa persona también deberá someterse a una evaluación de detección para determinar si es lo suficientemente saludable como para donar.

Si es tu hijo quien tiene una enfermedad renal, el trasplante puede ser el mejor tratamiento para él. El trasplante permite que los niños y adultos jóvenes se desarrollen de la manera más normal posible durante sus años formativos. Si tu hijo tiene una enfermedad renal, deberías considerar seriamente la posibilidad de realizar una evaluación de trasplante para él o ella.

Si el proceso de evaluación muestra que un trasplante es adecuado para ti o tu hijo, el siguiente paso es obtener un riñón adecuado. (Consulta "Encontrar un Riñón" a continuación.)"


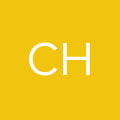


1. **You**

Please translate to Spanish: "What happens during kidney transplant surgery? The operation to place a donated kidney takes about four hours. The donated kidney is placed into your lower abdomen (belly), where it’s easiest to connect it to your important blood vessels and bladder. You may be surprised to learn that your own kidneys generally aren’t taken out when you get a transplant. The surgeon leaves them where they are unless there is a medical reason to remove them. After surgery, you’ll be sore at first, but you should be out of bed in a day or so, and home from the hospital within a week. If the kidney came from a living donor, it should start to work very quickly. A kidney from a deceased donor can take longer to start working—two to four weeks or more. This is called “delayed graft function”. If that happens, you may need dialysis until the kidney begins to work. After surgery, you’ll be taught about the medicines you’ll have to take and their side effects. You’ll also learn about how your diet may change after transplant. If you’ve been on dialysis, you’ll find that there are fewer restrictions on what you can eat and drink, which is one of the benefits of a transplant. You will want to eat a healthy diet and exercise to take good care of your body and your new kidney."

ChatGPT

**ChatGPT**

"¿Qué sucede durante la cirugía de trasplante de riñón? La operación para colocar un riñón donado dura aproximadamente cuatro horas. El riñón donado se coloca en la parte inferior de tu abdomen, donde es más fácil conectarlo a tus importantes vasos sanguíneos y vejiga. Puede que te sorprenda saber que por lo general no se extraen tus propios riñones cuando recibes un trasplante. El cirujano los deja en su lugar a menos que haya una razón médica para quitarlos.

Después de la cirugía, es probable que sientas dolor al principio, pero deberías poder levantarte de la cama en uno o dos días y regresar a casa desde el hospital en una semana. Si el riñón proviene de un donante vivo, debería comenzar a funcionar muy rápidamente. Un riñón de un donante fallecido puede tardar más en comenzar a funcionar, de dos a cuatro semanas o más. A esto se le llama "función retardada del injerto". Si eso sucede, es posible que necesites diálisis hasta que el riñón comience a funcionar.

Después de la cirugía, te enseñarán acerca de los medicamentos que deberás tomar y sus efectos secundarios. También aprenderás cómo puede cambiar tu dieta después del trasplante. Si has estado en diálisis, notarás que hay menos restricciones en lo que puedes comer y beber, lo cual es uno de los beneficios del trasplante. Querrás mantener una dieta saludable y hacer ejercicio para cuidar bien de tu cuerpo y de tu nuevo riñón."


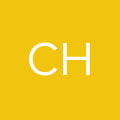


1. **You**

Please translate to Spanish: "What are anti-rejection medicines? Normally, your body fights off anything that isn’t part of itself, like germs and viruses. That system of protection is called your immune system. To stop your body from attacking or rejecting the donated kidney, you will have to take medicines to keep your immune system less active (called anti-rejection medicines or immunosuppressant medicines). You’ll need to take them as long as your new kidney is working. Without them, your immune system would see the donated kidney as “foreign,” and would attack and destroy it. Anti-rejection medicines can have some side effects. It is important to talk to your healthcare provider about them, so that you know what to expect. Fortunately, for most people, side effects are usually manageable. Changing the dose or type of medicine can often ease some of the side effects. Besides the immunosuppressive medicines, you will take other medicines as well. You will take medicines to protect you from infection, too. Most people find taking medicines a small trade for the freedom and quality of life that a successful transplant can provide."

ChatGPT

**ChatGPT**

"¿Qué son los medicamentos antirrechazo? Normalmente, tu cuerpo combate cualquier cosa que no forme parte de él, como gérmenes y virus. Ese sistema de protección se llama sistema inmunológico. Para evitar que tu cuerpo ataque o rechace el riñón donado, deberás tomar medicamentos que mantengan menos activo tu sistema inmunológico (llamados medicamentos antirrechazo o inmunosupresores). Tendrás que tomarlos mientras tu nuevo riñón esté funcionando. Sin estos medicamentos, tu sistema inmunológico vería al riñón donado como "extranjero" y lo atacaría y destruiría.

Los medicamentos antirrechazo pueden tener algunos efectos secundarios. Es importante hablar con tu proveedor de atención médica sobre ellos, para que sepas qué esperar. Afortunadamente, para la mayoría de las personas, los efectos secundarios suelen ser manejables. Cambiar la dosis o el tipo de medicamento a menudo puede aliviar algunos de los efectos secundarios.

Además de los medicamentos inmunosupresores, tomarás otros medicamentos también. Tomarás medicamentos para protegerte de las infecciones. La mayoría de las personas consideran que tomar medicamentos es un pequeño sacrificio por la libertad y la calidad de vida que un trasplante exitoso puede brindar."

2 / 2


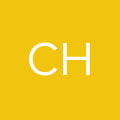


1. **You**

Please translate to Spanish: "Where do donated kidneys come from? A donated kidney may come from someone who died and donated a healthy kidney, called a deceased donor. Donated kidneys also can come from a living donor. This person may be a blood relative (like a brother or sister) or non-blood relative (like a husband or wife). They can also come from a friend or even a stranger. You do not need to have the same blood type as your donor to get a kidney if you are in a paired exchange program. Visit the Programs for Donor/Recipient Pairs with Incompatible Blood Types page to learn more about paired exchange programs. Kidneys from living or deceased donors both work well and can be good options, but kidneys from a living donor may last longer and are more likely to start working right away than a kidney from a deceased donor. Another plus is that when a kidney is donated by a living person, the operations can be scheduled at a convenient time for both the patient and the donor. Finding a living donor can also help you get a kidney faster since waiting for a deceased donor kidney can take many years."

ChatGPT

**ChatGPT**

"¿De dónde provienen los riñones donados? Un riñón donado puede provenir de alguien que falleció y donó un riñón sano, lo que se llama un donante fallecido. Los riñones donados también pueden provenir de un donante vivo. Esta persona puede ser un familiar de sangre (como un hermano o una hermana) o un no familiar de sangre (como un esposo o esposa). También pueden provenir de un amigo o incluso de un desconocido. No es necesario tener el mismo tipo de sangre que tu donante para recibir un riñón si estás en un programa de intercambio de parejas. Visita la página de Programas para Parejas de Donantes/Receptores con Tipos de Sangre Incompatibles para obtener más información sobre los programas de intercambio de parejas.

Los riñones de donantes vivos o fallecidos funcionan bien y pueden ser buenas opciones, pero los riñones de un donante vivo pueden durar más y tienen más probabilidades de comenzar a funcionar de inmediato que un riñón de un donante fallecido. Otra ventaja es que cuando un riñón es donado por una persona viva, las operaciones se pueden programar en un momento conveniente tanto para el paciente como para el donante. Encontrar un donante vivo también puede ayudarte a obtener un riñón más rápido, ya que esperar un riñón de un donante fallecido puede llevar muchos años."


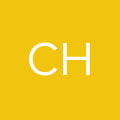


1. **You**

Please translate to Spanish: "How do I get a kidney from a deceased donor? To get a deceased donor kidney, you will be placed on a waiting list once you have been cleared for a transplant. It can take many years for a good donor kidney to be offered to you. From the time you go on the list until a kidney is found, you may have to be on some form of dialysis. While you’re waiting, you’ll need regular blood tests to make sure you are ready when a kidney is found. If you’re on dialysis, your center will make the arrangements for these tests. Your transplant center should know how to reach you at all times. Once a kidney become available, the surgery must be done as soon as possible."

ChatGPT

**ChatGPT**

"¿Cómo puedo obtener un riñón de un donante fallecido? Para obtener un riñón de un donante fallecido, serás colocado en una lista de espera una vez que hayas sido aprobado para un trasplante. Puede llevar muchos años que se te ofrezca un buen riñón de donante fallecido. Desde el momento en que entras en la lista hasta que se encuentra un riñón, es posible que tengas que recibir algún tipo de diálisis. Mientras esperas, necesitarás análisis de sangre regulares para asegurarte de estar listo cuando se encuentre un riñón. Si estás en diálisis, tu centro hará los arreglos para estos análisis. Tu centro de trasplantes debe saber cómo comunicarse contigo en todo momento. Una vez que esté disponible un riñón, la cirugía debe realizarse lo antes posible."


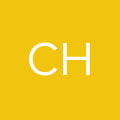


1. **You**

Please translate to Spanish: "How do I get a kidney from a living donor? To receive a kidney from a living donor, you must first be cleared by a transplant center after completing an evaluation. After that, the best place to start is by sharing your story with people you know and your community. Talk to your family and friends about your kidney failure and your need for a donor. To learn more about how to find a living donor, visit this link. "

ChatGPT

**ChatGPT**

"¿Cómo puedo obtener un riñón de un donante vivo? Para recibir un riñón de un donante vivo, primero debes ser aprobado por un centro de trasplantes después de completar una evaluación. Después de eso, el mejor lugar para comenzar es compartiendo tu historia con las personas que conoces y tu comunidad. Habla con tu familia y amigos sobre tu insuficiencia renal y tu necesidad de un donante. Para obtener más información sobre cómo encontrar un donante vivo, visita este enlace."


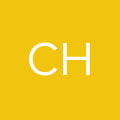


1. **You**

Please translate to Spanish: "Are there downsides to living donation? A healthy person who donates a kidney can live a normal life with the one kidney that is left, but a kidney donor must undergo surgery to remove a kidney and give it to the recipient. The operation is major surgery for the donor, as well as the recipient. As in any operation, there are some risks that you will want to consider , including surgery risks and long-term risks. The donor will need time to recover before returning to work and other activities. However, recent advances in surgery (often called minimally invasive or laparoscopic surgery) allow for very small incisions. This means shorter hospital stays and recovery time, less pain, and a quicker return to usual activities. Living donors often experience positive feelings about their courageous gift. Learn more about living donation here. "

ChatGPT

**ChatGPT**

"¿Existen desventajas en la donación en vida? Una persona sana que dona un riñón puede llevar una vida normal con el riñón que le queda, pero un donante de riñón debe someterse a una cirugía para extraer un riñón y dárselo al receptor. La operación es una cirugía importante tanto para el donante como para el receptor. Como en cualquier operación, existen algunos riesgos que querrás considerar, incluyendo riesgos quirúrgicos y riesgos a largo plazo. El donante necesitará tiempo para recuperarse antes de volver al trabajo y otras actividades. Sin embargo, avances recientes en cirugía (a menudo llamada cirugía mínimamente invasiva o laparoscópica) permiten incisiones muy pequeñas. Esto significa estancias hospitalarias más cortas y menos tiempo de recuperación, menos dolor y un retorno más rápido a las actividades habituales. Los donantes en vida a menudo experimentan sentimientos positivos acerca de su valioso regalo. Obtén más información sobre la donación en vida aquí."


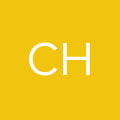


1. **You**

Please translate to Spanish: "What are the financial costs to the living donor? The surgery and evaluation are covered by Medicare or the recipient’s insurance. The living donor will not pay for anything related to the surgery. However, neither Medicare nor insurance covers time off from work, travel expenses, lodging, or other incidentals. The National Living Donor Assistance Program (www.livingdonorassistance.org) or other programs may help cover travel and lodging costs. Donors may be eligible for sick leave, state disability, and benefits under the federal Family Medical Leave Act. In addition, federal employees, some state employees, and certain other workers may be eligible for 30 days paid leave."

ChatGPT

**ChatGPT**

"¿Cuáles son los costos financieros para el donante en vida? La cirugía y la evaluación están cubiertas por Medicare o el seguro del receptor. El donante en vida no pagará nada relacionado con la cirugía. Sin embargo, ni Medicare ni el seguro cubren el tiempo fuera del trabajo, los gastos de viaje, alojamiento u otros gastos incidentales. El Programa Nacional de Asistencia para Donantes en Vida (www.livingdonorassistance.org) u otros programas pueden ayudar a cubrir los costos de viaje y alojamiento.

Los donantes pueden ser elegibles para licencia por enfermedad, discapacidad estatal y beneficios bajo la Ley Federal de Licencia Médica Familiar. Además, los empleados federales, algunos empleados estatales y ciertos otros trabajadores pueden ser elegibles para 30 días de licencia pagada."


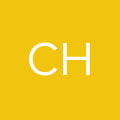


1. **You**

Please translate to Spanish: "What happens after I go home? Once you are home from the hospital, the most important work begins—the follow-up. For your transplant to be successful, you will have regular checkups, especially during the first year. At first, you may need blood tests several times a week, then once a week, then less often. As times passes, if you are doing well, you’ll need fewer checkups, but enough to make sure that your kidney is working well and that you have the right amount of anti-rejection medication in your body."

ChatGPT

**ChatGPT**

"¿Qué sucede después de regresar a casa? Una vez que estés en casa después del hospital, comienza la parte más importante: el seguimiento. Para que tu trasplante sea exitoso, tendrás revisiones periódicas, especialmente durante el primer año. Al principio, es posible que necesites análisis de sangre varias veces a la semana, luego una vez a la semana, y después con menos frecuencia. Con el tiempo, si estás progresando bien, necesitarás menos revisiones, pero las suficientes para asegurarte de que tu riñón esté funcionando correctamente y de que tengas la cantidad adecuada de medicamentos antirrechazo en tu cuerpo."


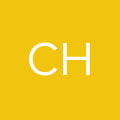


1. **You**

Please translate to Spanish: "What if my body tries to reject the new kidney? One thing that you and your healthcare team will watch for is acute rejection, which means that your body is suddenly “attacking” or trying to reject the transplanted kidney. A rejection episode may not have any clear signs or symptoms. That is why it is so important to have regular blood tests to check how well your kidney is working. Even though rejection can happen without symptoms, let your healthcare team know if you notice you are having fevers, peeing less or less often, swelling, weight gain, or pain over your kidney. The chances of having a rejection episode are highest right after your surgery. The longer you have the kidney, the lower the chance that this will happen. Unfortunately, sometimes a rejection episode happens even if you’re doing everything you’re supposed to do. Sometimes the body just doesn’t accept the transplanted kidney. But even if a rejection episode happens, there are many ways to treat it so you do not lose your transplant. Letting your transplant team know right away that you think you have symptoms of rejection is very important so they can treat it and help keep your transplanted kidney, and you, healthy."

ChatGPT

**ChatGPT**

"¿Qué sucede si mi cuerpo intenta rechazar el nuevo riñón? Una cosa que tú y tu equipo de atención médica vigilarán es el rechazo agudo, lo que significa que tu cuerpo está repentinamente "atacando" o intentando rechazar el riñón trasplantado. Un episodio de rechazo puede no tener signos o síntomas claros. Por eso es tan importante realizar análisis de sangre regulares para verificar qué tan bien está funcionando tu riñón. Aunque el rechazo puede ocurrir sin síntomas, debes informar a tu equipo de atención médica si notas que tienes fiebre, orinas menos o con menos frecuencia, hinchazón, aumento de peso o dolor sobre el riñón.

Las posibilidades de tener un episodio de rechazo son más altas justo después de la cirugía. Cuanto más tiempo tengas el riñón, menor será la probabilidad de que esto ocurra. Desafortunadamente, a veces ocurre un episodio de rechazo incluso si estás haciendo todo lo que debes hacer. A veces, el cuerpo simplemente no acepta el riñón trasplantado. Pero incluso si ocurre un episodio de rechazo, existen muchas formas de tratarlo para que no pierdas tu trasplante. Informar de inmediato a tu equipo de trasplante si crees que tienes síntomas de rechazo es muy importante para que puedan tratarlo y ayudar a mantener tu riñón trasplantado, y a ti, saludables."


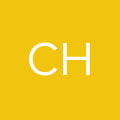


1. **You**

Please translate to Spanish: "How often does rejection happen? Rejections happen much less often than they used to. That’s because there have been many improvements in immunosuppressive medicines. However, the risk of rejection is different for every person. For most people, rejection can be stopped with special anti-rejection medicines. It’s very important to have regular checkups and tests to see how well your kidney is working, and make sure you are not having rejection."

ChatGPT

**ChatGPT**

"¿Con qué frecuencia ocurre el rechazo? El rechazo ocurre mucho menos frecuentemente de lo que solía ocurrir. Esto se debe a que ha habido muchas mejoras en los medicamentos inmunosupresores. Sin embargo, el riesgo de rechazo es diferente para cada persona. Para la mayoría de las personas, el rechazo puede detenerse con medicamentos especiales antirrechazo. Es muy importante tener revisiones y pruebas regulares para verificar qué tan bien está funcionando tu riñón y asegurarse de que no estás teniendo un rechazo."


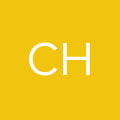


1. **You**

Please translate to Spanish: "When can I return to work? How soon you can return to work depends on your recovery, the kind of work you do, and your other medical conditions. Many people can return to work eight weeks or more after their transplant, but sometimes it can be sooner or later. Your transplant team will help you decide when you can go back to work."

ChatGPT

**ChatGPT**

"¿Cuándo puedo volver al trabajo? Cuánto tiempo te llevará volver al trabajo depende de tu recuperación, del tipo de trabajo que hagas y de tus otras condiciones médicas. Muchas personas pueden volver al trabajo ocho semanas o más después de su trasplante, pero a veces puede ser antes o después. Tu equipo de trasplante te ayudará a decidir cuándo puedes regresar al trabajo."


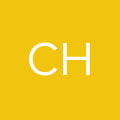


1. **You**

Please translate to Spanish: "How will a transplant affect my sex life? Can I still have children? People who have not had satisfactory sexual relations due to kidney disease may notice an improvement as they begin to feel better. In addition, fertility (the ability to conceive and have children) tends to increase. Men who have had a kidney transplant have fathered healthy children, and women with kidney transplants have had successful pregnancies. Talk to your healthcare practitioner when considering having a child. Women should avoid becoming pregnant too soon after a transplant. Most centers want women to wait a year or more. All pregnancies should be planned together with your care team. Certain medications can harm a developing baby, so they must be stopped at least six weeks before trying to get pregnant. Birth control or family planning counseling may be helpful. It’s important to protect yourself against sexually transmitted diseases (STDs). Be sure to use protection during sexual activity."

ChatGPT

**ChatGPT**

"¿Cómo afectará el trasplante a mi vida sexual? ¿Puedo seguir teniendo hijos? Las personas que no han tenido relaciones sexuales satisfactorias debido a la enfermedad renal pueden notar una mejora a medida que comienzan a sentirse mejor. Además, la fertilidad (la capacidad de concebir y tener hijos) tiende a aumentar. Los hombres que han tenido un trasplante de riñón han tenido hijos sanos, y las mujeres con trasplantes de riñón han tenido embarazos exitosos. Habla con tu profesional de la salud cuando estés considerando tener un hijo.

Las mujeres deben evitar quedar embarazadas demasiado pronto después de un trasplante. La mayoría de los centros quieren que las mujeres esperen un año o más. Todos los embarazos deben planificarse junto con tu equipo de atención. Ciertos medicamentos pueden dañar a un bebé en desarrollo, por lo que deben suspenderse al menos seis semanas antes de intentar quedar embarazada. El uso de anticonceptivos o el asesoramiento sobre planificación familiar pueden ser útiles.

Es importante protegerse contra las enfermedades de transmisión sexual (ETS). Asegúrate de usar protección durante la actividad sexual."


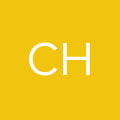


1. **You**

Please translate to Spanish: "Will I need to follow a special diet? In general, transplant recipients should eat a heart-healthy diet (low fat, low salt) and drink plenty of fluids. If you have diabetes or other health problems, you may still have some dietary restrictions. A dietitian can help you plan meals that are right for you."

ChatGPT

**ChatGPT**

"¿Necesitaré seguir una dieta especial? En general, los receptores de trasplantes deben seguir una dieta saludable para el corazón (baja en grasa, baja en sal) y beber suficientes líquidos. Si tienes diabetes u otros problemas de salud, es posible que aún debas tener algunas restricciones dietéticas. Un dietista puede ayudarte a planificar comidas que sean adecuadas para ti."


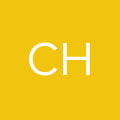


1. **You**

Please translate to Spanish: "What else can I do? You should learn as much as you can by reading and talking to your healthcare team, as well as patients who already have kidney transplants."

ChatGPT

**ChatGPT**

"¿Qué más puedo hacer? Deberías aprender todo lo que puedas leyendo y hablando con tu equipo de atención médica, así como con pacientes que ya tienen trasplantes de riñón."

RECIPIENT TRANSLATION SPANISH
CHAT GPT 4.0

**OPTN**


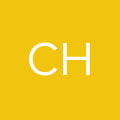


1. **You**

Please translate to Spanish: "How does a kidney transplant differ from dialysis? With dialysis treatment, a machine filters waste and extra fluid out of your blood the way your kidneys normally would. Many people undergo dialysis while awaiting a kidney transplant. Dialysis only filters the blood during a treatment session, not in the constant way a kidney does. For this reason, dialysis patients usually have diet and fluid restrictions. Dialysis also requires an access site – a vein or an implanted device where the blood can be collected and filtered outside the body. A kidney transplant is a surgical operation to give a functioning human kidney to someone whose kidneys have stopped working or are close to failing. The functioning kidney is removed from either a living donor or someone who has recently passed away. A transplanted kidney performs all the functions of a kidney a person has from birth. It constantly filters blood for waste and excess fluid. Most kidney transplant recipients do not have to limit their diet and fluid intake, but they do need to take medicine on a daily basis to help keep their transplant functioning."

ChatGPT

**ChatGPT**

"¿En qué se diferencia un trasplante de riñón de la diálisis? Con el tratamiento de diálisis, una máquina filtra los desechos y el exceso de líquido de la sangre de la manera en que lo harían normalmente los riñones. Muchas personas se someten a diálisis mientras esperan un trasplante de riñón.

La diálisis solo filtra la sangre durante una sesión de tratamiento, no de manera constante como lo hace un riñón. Por esta razón, los pacientes de diálisis generalmente tienen restricciones dietéticas y de líquidos. La diálisis también requiere un sitio de acceso, una vena o un dispositivo implantado donde se pueda recolectar y filtrar la sangre fuera del cuerpo.

Un trasplante de riñón es una operación quirúrgica para dar un riñón humano funcional a alguien cuyos riñones han dejado de funcionar o están a punto de fallar. El riñón funcional se extrae de un donante vivo o de alguien que ha fallecido recientemente.

Un riñón trasplantado realiza todas las funciones de un riñón que una persona tiene desde el nacimiento. Filtra constantemente la sangre para eliminar desechos y exceso de líquido. La mayoría de los receptores de trasplante de riñón no tienen que limitar su dieta y la ingesta de líquidos, pero necesitan tomar medicamentos a diario para ayudar a mantener su trasplante funcionando."


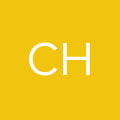


1. **You**

Please translate to Spanish: "What do I need to do to be considered for a transplant? To be considered for a kidney transplant, a transplant program must evaluate and accept you. You are not automatically listed for a transplant just because you begin dialysis or have some contact with a transplant program. Each transplant program makes its own decision about whether to accept someone for a transplant. The transplant team at each program has its own standards for accepting candidates. Each team may view the same facts and information different ways and make different decisions about listing a person for a transplant. Therefore, if one program is not willing to accept you as a candidate, a different program may accept you. Here are some common questions about what is involved with evaluation and testing, as well as factors that affect whether someone may be accepted for a transplant listing."

ChatGPT

**ChatGPT**

"¿Qué necesito hacer para ser considerado para un trasplante? Para ser considerado para un trasplante de riñón, un programa de trasplante debe evaluarte y aceptarte. No estás automáticamente en la lista para un trasplante solo porque comiences la diálisis o tengas algún contacto con un programa de trasplante.

Cada programa de trasplante toma su propia decisión sobre si aceptar a alguien para un trasplante. El equipo de trasplante de cada programa tiene sus propios estándares para aceptar candidatos. Cada equipo puede interpretar los mismos hechos e información de diferentes maneras y tomar diferentes decisiones sobre incluir a una persona en la lista para un trasplante. Por lo tanto, si un programa no está dispuesto a aceptarte como candidato, otro programa diferente puede aceptarte.

Aquí hay algunas preguntas comunes sobre lo que implica la evaluación y las pruebas, así como los factores que afectan si alguien puede ser aceptado para la lista de trasplantes."


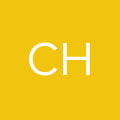


1. **You**

Please translate to Spanish: "Do I need a doctor’s referral, or can I contact the transplant program myself? You can contact the hospital yourself. You don’t need a doctor’s referral, but your doctor may have test results and medical history that will make it easier for the transplant team to evaluate you."

ChatGPT

**ChatGPT**

"Puedes contactar al hospital tú mismo. No necesitas una referencia médica, pero tu médico puede tener resultados de pruebas e historial médico que facilitarán la evaluación por parte del equipo de trasplante."


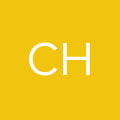


1. **You**

Please translate to Spanish: "Can I be evaluated for a transplant before I need dialysis? Yes. A standard test to assess your kidney function is your GFR (glomerular filtration rate). An ideal time to start the evaluation is when your GFR is between 25 and 30. Even if you haven’t reached end-stage kidney failure, starting the discussion early may give you time to consider your transplant options before you need dialysis."

ChatGPT

**ChatGPT**

"Sí. Una prueba estándar para evaluar la función de tus riñones es tu tasa de filtración glomerular (GFR, por sus siglas en inglés). Un momento ideal para comenzar la evaluación es cuando tu GFR está entre 25 y 30. Incluso si no has alcanzado la insuficiencia renal en etapa terminal, comenzar la discusión temprano puede darte tiempo para considerar tus opciones de trasplante antes de que necesites diálisis."


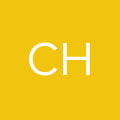


1. **You**

Please translate to Spanish: "What kinds of medical tests are done in an evaluation? The transplant team will do a number of medical tests. These include tests of your kidney function and other vital organs, screening for other diseases and medical conditions, and testing of your blood and genetic immune system markers to match you with compatible kidney offers. If you are on dialysis, the program also will want details about your dialysis treatment. If the program lists you for a transplant, it will also repeat some tests on a regular basis while you are on the waitlist."

ChatGPT

**ChatGPT**

"El equipo de trasplante realizará una serie de pruebas médicas. Estas incluyen pruebas de la función renal y otros órganos vitales, pruebas de detección de otras enfermedades y condiciones médicas, y pruebas de tu sangre y marcadores inmunológicos genéticos para emparejarte con ofertas de riñón compatibles. Si estás en diálisis, el programa también querrá detalles sobre tu tratamiento de diálisis. Si el programa te incluye en la lista para un trasplante, también repetirá algunas pruebas de manera regular mientras estés en la lista de espera."


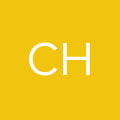


1. **You**

Please translate to Spanish: "How old is too old to be transplanted? There is no standard age limit to be transplanted. Each transplant program sets its own practice. For example, one program may not accept anyone older than 80 years of age; another may have no age limit. Most transplant candidates nationwide are age 50 or older, and many transplant programs often accept patients age 65 and older."

ChatGPT

**ChatGPT**

"No hay un límite de edad estándar para ser trasplantado. Cada programa de trasplante establece su propia práctica. Por ejemplo, un programa puede no aceptar a nadie mayor de 80 años; otro puede no tener límite de edad. La mayoría de los candidatos a trasplante a nivel nacional tienen 50 años o más, y muchos programas de trasplante a menudo aceptan pacientes de 65 años o más."


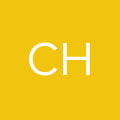


1. **You**

Please translate to Spanish: "What medical conditions might rule me out for a transplant listing? A few medical conditions might rule out a transplant. The transplant team will discuss these with you when you start your evaluation. In general, health issues that may keep you from having surgery or taking long-term medication may be reasons not to list you. These can include heart or lung conditions, current or recent cancer diagnosis, or morbid obesity. Depending on the medical issues involved, the program may have another specialist examine you and advise whether transplantation would be a safe option. In some cases, even if a transplant program doesn’t accept you immediately for a transplant, it may reconsider you later if your condition improves (for example, you lose weight as directed, or your cancer goes into remission). You also might consider applying to a different transplant center, since each hospital has its own criteria for accepting transplant candidates."

ChatGPT

**ChatGPT**

"Algunas condiciones médicas podrían descartar un trasplante. El equipo de trasplante discutirá estas contigo cuando comiences tu evaluación.

En general, problemas de salud que puedan impedirte someterte a una cirugía o tomar medicamentos a largo plazo pueden ser razones para no incluirte en la lista. Estos pueden incluir condiciones cardíacas o pulmonares, un diagnóstico de cáncer actual o reciente, o obesidad mórbida. Dependiendo de los problemas médicos involucrados, el programa puede tener otro especialista que te examine y aconseje si la trasplantación sería una opción segura.

En algunos casos, incluso si un programa de trasplante no te acepta inmediatamente para un trasplante, puede reconsiderarte más tarde si tu condición mejora (por ejemplo, pierdes peso según lo indicado, o tu cáncer entra en remisión). También podrías considerar solicitar en un centro de trasplante diferente, ya que cada hospital tiene sus propios criterios para aceptar candidatos a trasplante."


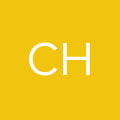


1. **You**

Please translate to Spanish: "Does a history of drug or alcohol abuse affect a transplant decision? A past history of drug or alcohol abuse may not rule out a person who has ended use and/or shown progress in treatment. During your evaluation, a social worker will meet with you to help the transplant team assess how your current behavior may affect your ability to do well with a transplant in the long term."

ChatGPT

**ChatGPT**

"¿Un historial de abuso de drogas o alcohol afecta la decisión de un trasplante? Un historial pasado de abuso de drogas o alcohol puede no descartar a una persona que ha terminado su uso y/o ha mostrado progreso en el tratamiento. Durante tu evaluación, un trabajador social se reunirá contigo para ayudar al equipo de trasplante a evaluar cómo tu comportamiento actual puede afectar tu capacidad para tener éxito con un trasplante a largo plazo."


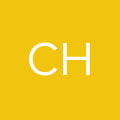


1. **You**

Please translate to Spanish: "Do I need medical insurance at the time I get evaluated? It’s important for the long-term success of your transplant for you to have insurance or other financial resources to cover needed expenses. Even after the transplant operation, you will take anti-rejection drugs and need follow-up medical care as long as you have a transplant. Every transplant program has a social worker or financial coordinator who can advise you on insurance or funding options"

ChatGPT

**ChatGPT**

"¿Necesito tener un seguro médico en el momento de mi evaluación? Es importante para el éxito a largo plazo de tu trasplante que tengas un seguro u otros recursos financieros para cubrir los gastos necesarios. Incluso después de la operación de trasplante, tomarás medicamentos anti-rechazo y necesitarás atención médica de seguimiento mientras tengas un trasplante. Cada programa de trasplante tiene un trabajador social o coordinador financiero que puede asesorarte sobre opciones de seguro o financiamiento."


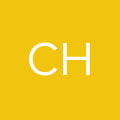


1. **You**

Please translate to Spanish: "Do I need to have a stable housing situation to be evaluated or listed for a transplant? It is best if you have a stable housing situation, so the transplant team can contact you for appointments and any potential organ offer after you are listed for transplant. A social worker with the transplant program may be able to help you if you don’t have a permanent place to stay."

ChatGPT

**ChatGPT**

"¿Necesito tener una situación de vivienda estable para ser evaluado o incluido en la lista para un trasplante? Es mejor si tienes una situación de vivienda estable, para que el equipo de trasplante pueda contactarte para citas y cualquier oferta de órgano potencial después de que estés en la lista para un trasplante. Un trabajador social del programa de trasplante puede ayudarte si no tienes un lugar permanente donde alojarte."


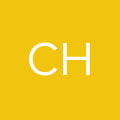


1. **You**

Please translate to Spanish: "What else will the transplant team want to know about me? You will talk with a psychologist and/or a social worker, who are members of the transplant team. The team will want to be sure you can understand and follow medical instructions. They also will want to know what social support you have (family, friends and/or caregivers) to help you with tasks such as attending appointments and taking your medications. This is important to help ensure you will do well long after the transplant operation. If you are accepted for a transplant, the transplant team will need emergency contact information for you and for any backup person you name. This helps them contact you quickly for matching kidney offers. You also will need to set and keep appointments on a regular basis to allow the transplant staff to check your condition while you are waiting for a transplant."

ChatGPT

**ChatGPT**

"¿Qué más querrá saber el equipo de trasplante sobre mí? Hablarás con un psicólogo y/o un trabajador social, quienes son miembros del equipo de trasplante. El equipo querrá estar seguro de que puedes entender y seguir las instrucciones médicas. También querrán saber qué apoyo social tienes (familia, amigos y/o cuidadores) para ayudarte con tareas como asistir a citas y tomar tus medicamentos. Esto es importante para ayudar a asegurar que te vaya bien mucho después de la operación de trasplante. Si eres aceptado para un trasplante, el equipo de trasplante necesitará información de contacto de emergencia para ti y para cualquier persona de respaldo que nombres. Esto les ayuda a contactarte rápidamente para ofertas de riñón compatibles. También necesitarás establecer y mantener citas de manera regular para permitir que el personal de trasplante revise tu condición mientras esperas un trasplante."


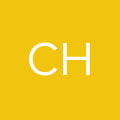


1. **You**

Please translate to Spanish: "Can I list at more than one transplant program at the same time? It is possible to list at multiple transplant programs, but each program would decide on its own whether to accept and list you. You would need to meet all the requirements the transplant team sets at any program where you are listed. Transplant candidates listed at a program close to the organ donor hospital are usually considered for matching offers before those at more distant hospitals. Listing at programs in different areas of the country might help someone find a match more quickly."

ChatGPT

**ChatGPT**

"¿Puedo estar en la lista de más de un programa de trasplante al mismo tiempo? Es posible estar en la lista de varios programas de trasplante, pero cada programa decidirá por sí mismo si aceptarte y listarte. Necesitarías cumplir con todos los requisitos que el equipo de trasplante establezca en cualquier programa donde estés en la lista. Los candidatos a trasplante listados en un programa cerca del hospital donante de órganos generalmente se consideran para ofertas de emparejamiento antes que aquellos en hospitales más lejanos. Estar en la lista de programas en diferentes áreas del país podría ayudar a alguien a encontrar una coincidencia más rápidamente."


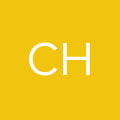


1. **You**

Please translate to Spanish: "How does the transplant process work? Kidneys can be transplanted from living or deceased donors. Below are common questions relating to living donor transplants."

ChatGPT

**ChatGPT**

"¿Cómo funciona el proceso de trasplante? Los riñones pueden ser trasplantados de donantes vivos o fallecidos. A continuación, se presentan preguntas comunes relacionadas con los trasplantes de donantes vivos."


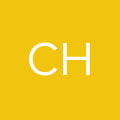


1. **You**

Please translate to Spanish: "Who can be a living donor? A living donor may be a family member, friend, coworker, neighbor, or even a complete stranger, as long as they are a medical match for you. Your transplant team can advise you how to discuss living donation with people who might be interested. All living donors must make a free and independent choice. They must understand there are medical risks to donating a kidney. -Life expectancy - Donating a kidney is major surgery but the procedure has not been shown to reduce the donor’s life expectancy. -Surgical Complications – Donors face possible post-operative complications such as bleeding, wound infection, fever, etc., but most complications are generally short- term and can be treated with quality medical care. -Recovery Time - Recovery after surgery should take between three and six weeks. You may need to stay in the hospital for up to seven days. Your doctor or surgeon will discuss the success of the surgery and any follow-up treatments you may need. Living donors will have a number of medical tests to help ensure they are healthy enough to donate. They also will talk to a psychologist and/or social worker to help the transplant team make sure they have realistic expectations and reasons for donation.

ChatGPT

**ChatGPT**

"¿Quién puede ser un donante vivo? Un donante vivo puede ser un miembro de la familia, un amigo, un compañero de trabajo, un vecino, o incluso un completo desconocido, siempre y cuando sea médicamente compatible contigo. Tu equipo de trasplante puede asesorarte sobre cómo discutir la donación en vida con personas que podrían estar interesadas. Todos los donantes vivos deben tomar una decisión libre e independiente. Deben entender que hay riesgos médicos al donar un riñón.

- Expectativa de vida - Donar un riñón es una cirugía mayor, pero el procedimiento no ha demostrado reducir la expectativa de vida del donante.
- Complicaciones quirúrgicas – Los donantes enfrentan posibles complicaciones postoperatorias como sangrado, infección de la herida, fiebre, etc., pero la mayoría de las complicaciones son generalmente a corto plazo y pueden ser tratadas con atención médica de calidad.
- Tiempo de recuperación - La recuperación después de la cirugía debería tomar entre tres y seis semanas. Puede que necesites permanecer en el hospital hasta siete días. Tu médico o cirujano discutirá el éxito de la cirugía y cualquier tratamiento de seguimiento que puedas necesitar. Los donantes vivos tendrán una serie de pruebas médicas para ayudar a asegurar que están lo suficientemente sanos para donar. También hablarán con un psicólogo y/o trabajador social para ayudar al equipo de trasplante a asegurarse de que tienen expectativas realistas y razones para la donación."


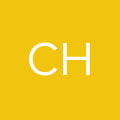


1. **You**

Please translate to Spanish: "What is kidney paired donation? If you have a potential donor who is not a biologic match with you (such as having an incompatible blood type), but may match with someone else, kidney paired donation may be an option. In kidney paired donation, information about potential living donors and recipients is matched in a computer to find successful donor-recipient combinations. So the person who wanted to donate to you can donate to a different patient, and that patient’s intended donor can donate a kidney to you. Kidney paired donation may often help transplant candidates who are very hard to match."

ChatGPT

**ChatGPT**

"¿Qué es la donación renal apareada? Si tienes un donante potencial que no es compatible biológicamente contigo (como tener un tipo de sangre incompatible), pero puede coincidir con otra persona, la donación renal apareada puede ser una opción.

En la donación renal apareada, la información sobre donantes y receptores vivos potenciales se empareja en una computadora para encontrar combinaciones exitosas de donante-receptor. Así, la persona que quería donarte puede donar a un paciente diferente, y el donante previsto de ese paciente puede donarte un riñón a ti. La donación renal apareada a menudo puede ayudar a candidatos a trasplante que son muy difíciles de emparejar."


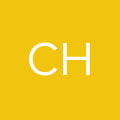


1. **You**

Please translate to Spanish: "How long does it take for a living donor transplant? That depends on a number of factors, but a wait of several weeks to a few months is common. Living donor transplants are routinely scheduled operations. The donation and transplant procedures are closely timed so that the transplant has the best chance of success."

ChatGPT

**ChatGPT**

"¿Cuánto tiempo lleva realizar un trasplante de donante vivo? Eso depende de varios factores, pero una espera de varias semanas a unos pocos meses es común. Los trasplantes de donantes vivos son operaciones programadas de manera rutinaria. Los procedimientos de donación y trasplante se coordinan de cerca para que el trasplante tenga la mejor oportunidad de éxito."


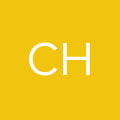


1. **You**

Please translate to Spanish: "How would I be matched for a kidney from a deceased donor? The computer database of the Organ Procurement and Transplantation Network (OPTN) matches medical information on all transplant candidates who are actively listed on the waitlist nationwide with medical information on deceased donors. The database produces a rank-ordered list for each donor offer that determines who will be offered the kidney. When a transplant team gets an organ offer for one of its patients, it reviews detailed medical information about the donor. The team will then decide whether to accept the offer for their candidate. If the offer is turned down for one candidate, it goes to the next person on the list. Kidneys that are likely to function for the longest amount of time are considered first for candidates who are likely to need them the longest. Other factors in matching include how closely the donor’s blood and tissue type matches the patient, how long the candidate has needed a kidney transplant, and how close the transplant hospital is to the donor hospital."

ChatGPT

**ChatGPT**

"¿Cómo sería emparejado para un riñón de un donante fallecido? La base de datos computarizada de la Red de Obtención y Trasplante de Órganos (OPTN, por sus siglas en inglés) empareja la información médica de todos los candidatos a trasplante que están activamente en la lista de espera a nivel nacional con la información médica de los donantes fallecidos. La base de datos produce una lista ordenada por rango para cada oferta de donante que determina a quién se le ofrecerá el riñón.

Cuando un equipo de trasplante recibe una oferta de órgano para uno de sus pacientes, revisa información médica detallada sobre el donante. El equipo entonces decidirá si acepta la oferta para su candidato. Si la oferta se rechaza para un candidato, pasa a la siguiente persona en la lista.

Los riñones que probablemente funcionarán durante el mayor tiempo posible se consideran primero para candidatos que probablemente los necesiten durante más tiempo. Otros factores en el emparejamiento incluyen cuán estrechamente coincide el tipo de sangre y tejido del donante con el paciente, cuánto tiempo ha necesitado el candidato un trasplante de riñón y qué tan cerca está el hospital de trasplante del hospital donante."


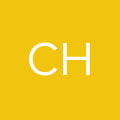


1. **You**

Please translate to Spanish: "How long is the wait for a deceased donor transplant? Unfortunately, there are not enough organs available for everyone who needs a kidney transplant. Waiting times for a deceased donor kidney transplant can vary greatly in different parts of the country, but waiting times of 4 to 5 years are common. Some people may wait much shorter or longer based on how well they match with most donors. Your transplant program can tell you more about how long you might expect to wait."

ChatGPT

**ChatGPT**

"¿Cuánto tiempo es la espera para un trasplante de donante fallecido? Desafortunadamente, no hay suficientes órganos disponibles para todos los que necesitan un trasplante de riñón. Los tiempos de espera para un trasplante de riñón de un donante fallecido pueden variar mucho en diferentes partes del país, pero tiempos de espera de 4 a 5 años son comunes. Algunas personas pueden esperar mucho menos o más tiempo en función de qué tan bien coinciden con la mayoría de los donantes. Tu programa de trasplante puede informarte más sobre cuánto tiempo podrías esperar."


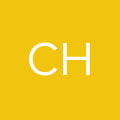


1. **You**

Please translate to Spanish: "What must I do after a transplant? A transplant is a treatment for kidney failure, not a cure. After a transplant, you will need ongoing treatment to stay healthy and keep your kidney working well. Your immune system will recognize the transplanted kidney as being “different.” It may try to attack (reject) the organ. To lower the chance of organ rejection, you will need to take medicine every day. Your transplant team wants to make sure your kidney is functioning properly and that you’re taking the right doses of medicine to prevent or treat organ rejection. As long as you have a functioning transplant, you will need to have regular medical tests and appointments. These tests and appointments are more frequent just after your transplant. If you do well in the longer term, you do not need to have them as often."

ChatGPT

**ChatGPT**

"¿Qué debo hacer después de un trasplante? Un trasplante es un tratamiento para la insuficiencia renal, no una cura. Después de un trasplante, necesitarás tratamiento continuo para mantenerte sano y mantener tu riñón funcionando bien.

Tu sistema inmunológico reconocerá el riñón trasplantado como "diferente". Puede intentar atacar (rechazar) el órgano. Para reducir la posibilidad de rechazo del órgano, necesitarás tomar medicamentos todos los días. Tu equipo de trasplante quiere asegurarse de que tu riñón está funcionando correctamente y que estás tomando las dosis correctas de medicamentos para prevenir o tratar el rechazo del órgano. Mientras tengas un trasplante funcionando, necesitarás someterte a pruebas médicas regulares y tener citas. Estas pruebas y citas son más frecuentes justo después de tu trasplante. Si te va bien a largo plazo, no necesitas tenerlas tan a menudo."

**NHS**


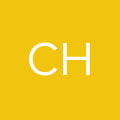


1. **You**

Please translate to Spanish: "What is the life expectancy of a kidney transplant patient? Life expectancy for patients with kidney disease is very variable depending on age and other medical issues. Please discuss this with the team caring for you."

ChatGPT

**ChatGPT**

"¿Cuál es la esperanza de vida de un paciente con trasplante de riñón? La esperanza de vida para los pacientes con enfermedad renal es muy variable dependiendo de la edad y otros problemas médicos. Por favor, discuta esto con el equipo que lo atiende."


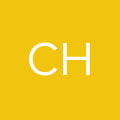


1. **You**

Please translate to Spanish: "What is the life expectancy of a transplanted kidney? On average, a kidney transplant from a living donor lasts for 20-25 years, while a kidney from a deceased donor lasts 15-20 years. This is very variable depending on the age of the donor and patient, and other medical issues. Please speak to your kidney care team for more personalised information. Some patients are able to have additional kidney transplants after this."

ChatGPT

**ChatGPT**

"¿Cuál es la esperanza de vida de un riñón trasplantado? En promedio, un trasplante de riñón de un donante vivo dura entre 20 y 25 años, mientras que un riñón de un donante fallecido dura entre 15 y 20 años.

Esto es muy variable dependiendo de la edad del donante y del paciente, y otros problemas médicos. Por favor, hable con su equipo de cuidado renal para obtener información más personalizada.

Algunos pacientes pueden recibir trasplantes de riñón adicionales después de esto."


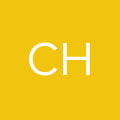


1. **You**

Please translate to Spanish: "How successful are kidney transplants in UK? For patients that receive a living donor kidney transplant, 99 out of 100 patients are alive after one year and 86 out of 100 are alive after ten years. For patients that receive a deceased donor kidney transplant, 96 to 97 out of 100 patients are alive after one year and 72 to 76 out of 100 are alive after ten years."

ChatGPT

**ChatGPT**

"¿Qué tan exitosos son los trasplantes de riñón en el Reino Unido? Para los pacientes que reciben un trasplante de riñón de un donante vivo, 99 de cada 100 pacientes están vivos después de un año y 86 de cada 100 están vivos después de diez años.

Para los pacientes que reciben un trasplante de riñón de un donante fallecido, de 96 a 97 de cada 100 pacientes están vivos después de un año y de 72 a 76 de cada 100 están vivos después de diez años."


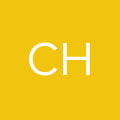


1. **You**

Please translate to Spanish: "What is the longest a transplanted kidney has lasted? Some patients have had a kidney transplant that has kept working for more than 40 years. Up-to-date records on the longest functioning kidney transplant are not kept."

ChatGPT

**ChatGPT**

"¿Cuál es el tiempo más largo que ha durado un riñón trasplantado? Algunos pacientes han tenido un trasplante de riñón que ha seguido funcionando durante más de 40 años. No se mantienen registros actualizados sobre el trasplante de riñón funcionando más tiempo."


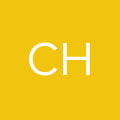


1. **You**

Please translate to Spanish: "What is the maximum age for a kidney transplant? All decisions on transplantation are made on a case-by-case basis. If you have concerns that you might be too old for a kidney transplant, please discuss this with your kidney care team."

ChatGPT

**ChatGPT**

"¿Cuál es la edad máxima para un trasplante de riñón? Todas las decisiones sobre el trasplante se toman caso por caso. Si tiene preocupaciones de que podría ser demasiado mayor para un trasplante de riñón, por favor discuta esto con su equipo de cuidado renal."


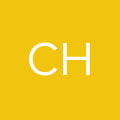


1. **You**

Please translate to Spanish: "How long is the wait for a kidney? The average wait for a deceased donor kidney transplant in the UK is 2-3 years. If you have a living donor, it usually takes just 3-6 months to have the required tests on you and your donor and to arrange the transplant."

ChatGPT

**ChatGPT**

"¿Cuánto tiempo se espera por un riñón? El tiempo de espera promedio para un trasplante de riñón de un donante fallecido en el Reino Unido es de 2 a 3 años.

Si tiene un donante vivo, generalmente solo toma de 3 a 6 meses realizar las pruebas necesarias en usted y su donante y organizar el trasplante."


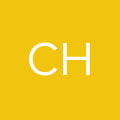


1. **You**

Please translate to Spanish: "How long will I need to be in hospital after a kidney transplant? If you’re receiving a kidney from a living donor – the usual stay is 4 to 6 days. If you’re receiving a kidney from a deceased donor - most patients stay in hospital for 6 to 10 days."

ChatGPT

**ChatGPT**

"¿Cuánto tiempo necesitaré estar en el hospital después de un trasplante de riñón? Si va a recibir un riñón de un donante vivo, la estancia habitual es de 4 a 6 días.

Si va a recibir un riñón de un donante fallecido, la mayoría de los pacientes permanecen en el hospital de 6 a 10 días."


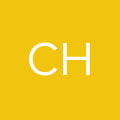


1. **You**

Please translate to Spanish: "How long does it take to recover from a kidney transplant? Everyone recovers at a different rate after a kidney transplant. For patients that worked before the transplant, they are usually back to work within 2-3 months of the transplant. Once the first few months after your operation have passed, it is likely that your kidney function will be stable and you won’t need to come to the transplant clinic as often. After the first 3-6 months, the risk of transplant rejection is lower and your medicines will usually reduce."

ChatGPT

**ChatGPT**

"¿Cuánto tiempo se tarda en recuperarse de un trasplante de riñón? Todos se recuperan a un ritmo diferente después de un trasplante de riñón. Para los pacientes que trabajaban antes del trasplante, generalmente vuelven al trabajo dentro de los 2 a 3 meses posteriores al trasplante. Una vez que hayan pasado los primeros meses después de su operación, es probable que su función renal sea estable y no necesite acudir a la clínica de trasplantes tan a menudo. Después de los primeros 3 a 6 meses, el riesgo de rechazo del trasplante es menor y sus medicamentos generalmente se reducirán."


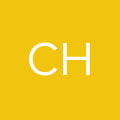


1. **You**

Please translate to Spanish: "Can I die from a kidney transplant? As with any major operation, there is a small risk of dying, but this is rare. The average risk of dying during the first few months after a kidney transplant is between 1-2 in 100 patients. However, this varies widely on a patient-by-patient basis."

ChatGPT

**ChatGPT**

"¿Puedo morir a causa de un trasplante de riñón? Como en cualquier operación mayor, existe un pequeño riesgo de morir, pero esto es raro. El riesgo promedio de morir durante los primeros meses después de un trasplante de riñón es de entre 1 y 2 de cada 100 pacientes. Sin embargo, esto varía ampliamente de un paciente a otro."


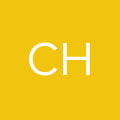


1. **You**

Please translate to Spanish: "Will my original kidney disease come back after the transplant? This can happen with some patients and is known as ‘recurrence’. Some kidney diseases can come back and affect the kidney transplant very quickly (days to weeks), but this is very rare. If this is a possibility, your kidney doctors and transplant surgeons will discuss this with you. If you have had difficult medical problems before you developed kidney failure, this may make it more difficult to have a transplant. You will have a number of different medical tests to see if a kidney transplant is the best treatment option for you."

ChatGPT

**ChatGPT**

"¿Volverá mi enfermedad renal original después del trasplante? Esto puede suceder con algunos pacientes y se conoce como 'recurrencia'.

Algunas enfermedades renales pueden regresar y afectar el trasplante de riñón muy rápidamente (días a semanas), pero esto es muy raro. Si esto es una posibilidad, sus médicos renales y cirujanos de trasplante lo discutirán con usted. Si ha tenido problemas médicos difíciles antes de desarrollar insuficiencia renal, esto puede dificultar tener un trasplante. Se le realizarán una serie de diferentes pruebas médicas para ver si un trasplante de riñón es la mejor opción de tratamiento para usted."


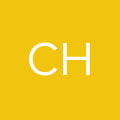


1. **You**

Please translate to Spanish: "Can I have another kidney transplant if my transplant stops working? Yes. It is possible to have multiple kidney transplants. You will need to either receive a kidney from a living donor or go back on the national transplant waiting list."

ChatGPT

**ChatGPT**

"Sí. Es posible tener múltiples trasplantes de riñón. Necesitará recibir un riñón de un donante vivo o volver a la lista de espera nacional de trasplantes."


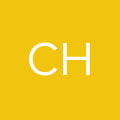


1. **You**

Please translate to Spanish: "Can I donate an organ if I’ve had a transplant? Yes, this is possible. Your general health will be taken into account. Please consider recording your wishes on organ donation in the national register.

ChatGPT

**ChatGPT**

"Sí, esto es posible. Se tendrá en cuenta su estado de salud general. Por favor, considere registrar sus deseos sobre la donación de órganos en el registro nacional."


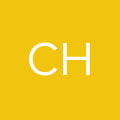


1. **You**

Please translate to Spanish: "How many kidney transplants are carried out per year in the UK? There were 2,868 adult kidney transplants performed in 2021/2022 in the UK."

ChatGPT

**ChatGPT**

"¿Cuántos trasplantes de riñón se realizan por año en el Reino Unido? Se realizaron 2,868 trasplantes de riñón en adultos en el año 2021/2022 en el Reino Unido."


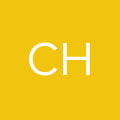


1. **You**

Please translate to Spanish: "How many people are waiting for kidney transplants in the UK? In the UK, around 5,000 people are in need of a kidney transplant."

ChatGPT

**ChatGPT**

"¿Cuántas personas están esperando trasplantes de riñón en el Reino Unido? En el Reino Unido, alrededor de 5,000 personas necesitan un trasplante de riñón."


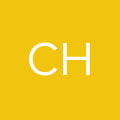


1. **You**

Please translate to Spanish: "How long is kidney transplant surgery? Kidney transplant surgery takes between 2-4 hours."

ChatGPT

**ChatGPT**

"¿Cuánto dura la cirugía de trasplante de riñón? La cirugía de trasplante de riñón dura entre 2 y 4 horas."

**NKF**


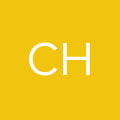


1. **You**

Please translate to Spanish: "What is a kidney transplant? When you get a kidney transplant, a healthy kidney is placed inside your body to do the work your own kidneys can no longer do. The healthy kidney can come from someone has died and chosen to donate, called a deceased donor, or from someone who has two healthy kidneys and chooses to donate one, called a living donor. A successful kidney transplant may allow you to live longer and to live the kind of life you were living before you got kidney disease. For many patients, there are fewer limits on what you can eat and drink, though you should follow a heart-healthy diet and maintain a healthy weight to help your new kidney last. Your health and energy should also improve. Studies show that people with kidney transplants live longer than those who remain on dialysis. Having a kidney transplant does not “cure” kidney disease. There are also risks, including the risks of surgery. After the transplant, you will need to take anti-rejection medicines for as long as your new kidney is working, which can have side effects. You will have a higher risk for infections and certain types of cancer. Although most transplants are successful and last for many years, how long they last can vary from one person to the next. Depending on your age, many people will need more than one kidney transplant during a lifetime."

ChatGPT

**ChatGPT**

"¿Qué es un trasplante de riñón?

Cuando te realizan un trasplante de riñón, se coloca un riñón sano dentro de tu cuerpo para realizar el trabajo que tus propios riñones ya no pueden hacer. El riñón sano puede provenir de alguien que ha fallecido y ha elegido donar, a quien se le llama donante fallecido, o de alguien que tiene dos riñones sanos y elige donar uno, a quien se le llama donante vivo.

Un trasplante de riñón exitoso puede permitirte vivir más tiempo y llevar el tipo de vida que tenías antes de padecer la enfermedad renal. Para muchos pacientes, hay menos limitaciones en lo que puedes comer y beber, aunque debes seguir una dieta saludable para el corazón y mantener un peso saludable para ayudar a que tu nuevo riñón dure. Tu salud y energía también deberían mejorar. Los estudios muestran que las personas con trasplantes de riñón viven más tiempo que aquellas que permanecen en diálisis.

Tener un trasplante de riñón no significa una “cura” para la enfermedad renal. También hay riesgos, incluyendo los riesgos de la cirugía. Después del trasplante, necesitarás tomar medicamentos anti-rechazo mientras tu nuevo riñón esté funcionando, lo que puede tener efectos secundarios. Tendrás un mayor riesgo de infecciones y ciertos tipos de cáncer.

Aunque la mayoría de los trasplantes son exitosos y duran muchos años, cuánto tiempo duran puede variar de una persona a otra. Dependiendo de tu edad, muchas personas necesitarán más de un trasplante de riñón durante su vida."


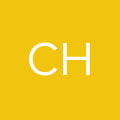


1. **You**

Please translate to Spanish: "What is a “preemptive” or “early” transplant? Getting a transplant before you need to start dialysis is called a preemptive transplant. It allows you to avoid dialysis altogether. Getting a transplant not long after kidneys fail (but with some time on dialysis) is referred to as an early transplant. Both have benefits. Some research shows that a pre-emptive or early transplant, with little or no time spent on dialysis, can lead to better long-term health. It may also allow you to keep working, save time and money, and have a better quality of life. If your doctor has told you that your kidney disease is progressing or that you are in a later stage of kidney disease, called Stage 3B, Stage 4, or Stage 5, it could be a good time to talk about kidney transplant."

ChatGPT

**ChatGPT**

"¿Qué es un trasplante "preventivo" o "temprano"?

Recibir un trasplante antes de necesitar iniciar la diálisis se llama trasplante preventivo. Esto te permite evitar la diálisis por completo. Recibir un trasplante poco después de que los riñones fallen (pero con algún tiempo en diálisis) se conoce como un trasplante temprano. Ambos tienen beneficios. Algunas investigaciones muestran que un trasplante preventivo o temprano, con poco o ningún tiempo en diálisis, puede conducir a una mejor salud a largo plazo. También puede permitirte seguir trabajando, ahorrar tiempo y dinero, y tener una mejor calidad de vida. Si tu médico te ha dicho que tu enfermedad renal está progresando o que estás en una etapa avanzada de la enfermedad renal, llamada Etapa 3B, Etapa 4 o Etapa 5, podría ser un buen momento para hablar sobre el trasplante de riñón."


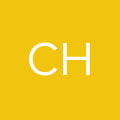


1. **You**

Please translate to Spanish: "Who can get a kidney transplant? Kidney patients of all ages—from children to seniors—are able to consider a transplant. For most people, getting a transplant can be a good treatment choice. Every person being considered for transplant will complete a full medical and psychosocial evaluation at a transplant center to make sure they are a good candidate. The evaluation helps find any problems, so they can be corrected before transplant. You must be healthy enough to have the operation. You must also be free from cancer and infection."

ChatGPT

**ChatGPT**

"¿Quién puede recibir un trasplante de riñón? Pacientes renales de todas las edades, desde niños hasta personas mayores, pueden considerar un trasplante. Para la mayoría de las personas, recibir un trasplante puede ser una buena opción de tratamiento.

Cada persona que se considere para un trasplante completará una evaluación médica y psicosocial completa en un centro de trasplantes para asegurarse de que es un buen candidato. La evaluación ayuda a encontrar cualquier problema, para que puedan ser corregidos antes del trasplante. Debes estar lo suficientemente saludable para someterte a la operación. También debes estar libre de cáncer e infecciones."


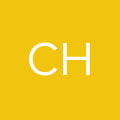


1. **You**

Please translate to Spanish: "What if I’m older or have other health problems? In many cases, people who are older or have other health conditions like diabetes can still have successful kidney transplants. Careful evaluation at a transplant center is needed to understand and deal with any special risks. You may be asked to do some things that can lessen certain risks and improve the chances of a successful transplant. For example, you may be asked to lose weight or quit smoking. Only a transplant center can decide if you are healthy enough to receive a kidney transplant. If you have diabetes, you may also be able to have a pancreas transplant. Ask your healthcare professional about getting a pancreas transplant along with a kidney transplant."

ChatGPT

**ChatGPT**

"¿Qué pasa si soy mayor o tengo otros problemas de salud?

En muchos casos, las personas mayores o con otras condiciones de salud, como la diabetes, aún pueden tener trasplantes de riñón exitosos. Se necesita una evaluación cuidadosa en un centro de trasplantes para entender y manejar cualquier riesgo especial. Puede que se te pida hacer algunas cosas que pueden disminuir ciertos riesgos y mejorar las posibilidades de un trasplante exitoso. Por ejemplo, se te puede pedir que pierdas peso o dejes de fumar. Solo un centro de trasplantes puede decidir si estás lo suficientemente saludable para recibir un trasplante de riñón.

Si tienes diabetes, también podrías ser candidato para un trasplante de páncreas. Pregunta a tu profesional de la salud sobre la posibilidad de obtener un trasplante de páncreas junto con un trasplante de riñón."


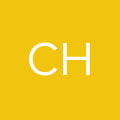


1. **You**

Please translate to Spanish: "How will I pay for a transplant? Medicare covers about 80% of the costs associated with an evaluation, transplant operation, follow-up care, and anti-rejection medicines. Private insurance through your job and state insurance programs may cover some costs as well. However, your post-transplant expenses may only be covered for a limited number of years. It’s important to discuss insurance coverage with your social worker and the transplant financial coordinator, who can answer your questions or direct you to others who can help. "

ChatGPT

**ChatGPT**

"¿Cómo pagaré por un trasplante?

Medicare cubre aproximadamente el 80% de los costos asociados con la evaluación, la operación de trasplante, el cuidado de seguimiento y los medicamentos anti-rechazo. El seguro privado a través de tu trabajo y los programas de seguro estatales también pueden cubrir algunos costos. Sin embargo, tus gastos post-trasplante pueden estar cubiertos solo por un número limitado de años. Es importante discutir la cobertura del seguro con tu trabajador social y el coordinador financiero del trasplante, quienes pueden responder tus preguntas o dirigirte a otras personas que puedan ayudar."


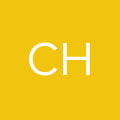


1. **You**

Please translate to Spanish: "How do I start the process of getting a kidney transplant? Ask your healthcare provider to refer you to a transplant center for an evaluation, or contact a transplant center in your area. Any kidney patient can ask for an evaluation, with or without a referral from their doctor."

ChatGPT

**ChatGPT**

"¿Cómo inicio el proceso para obtener un trasplante de riñón? Pídele a tu proveedor de atención médica que te derive a un centro de trasplantes para una evaluación, o contacta directamente un centro de trasplantes en tu área. Cualquier paciente renal puede solicitar una evaluación, con o sin una derivación de su médico."


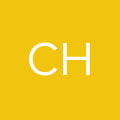


1. **You**

Please translate to Spanish: "How does the evaluation process work? The evaluation process for a transplant is very thorough. Your healthcare team will need to know a lot about you to help them—and you—decide if a transplant is right for you. Medical professionals will give you a complete physical exam, review your health records, and order a series of tests and X-rays to learn about your overall health. Everything that can affect how well you can handle a transplant will be checked. One thing you can do to speed the process is to get all the testing done as quickly as possible and stay in close contact with the transplant team. If you’re told you might not be right for a transplant, don’t be afraid to ask why. You can also consider doing an evaluation at another center if one center says no. Remember, being active in your own care is one of the best ways to stay healthy. If someone you know would like to donate a kidney to you, that person will also need to go through a screening evaluation to find out if he or she is healthy enough to donate. If it’s your child who has kidney disease, transplant can be the best treatment for them. Transplantation allows children and young adults to develop in as normal a way as possible in their formative years. If your child has kidney disease, you’ll want to give serious thought to getting a transplant evaluation for him or her. If the evaluation process shows that a transplant is right for you or your child, the next step is getting a suitable kidney. (See "Finding a Kidney" below.)"

ChatGPT

**ChatGPT**

"¿Cómo funciona el proceso de evaluación? El proceso de evaluación para un trasplante es muy exhaustivo. Tu equipo de atención médica necesitará saber mucho sobre ti para ayudarles a ellos y a ti a decidir si un trasplante es adecuado para ti. Los profesionales médicos te realizarán un examen físico completo, revisarán tus registros de salud y ordenarán una serie de pruebas y radiografías para conocer tu estado de salud general. Se verificará todo lo que pueda afectar cuán bien puedas manejar un trasplante.

Una cosa que puedes hacer para acelerar el proceso es completar todas las pruebas lo más rápido posible y mantener un contacto cercano con el equipo de trasplante. Si te dicen que podrías no ser adecuado para un trasplante, no tengas miedo de preguntar por qué. También puedes considerar hacer una evaluación en otro centro si uno dice que no. Recuerda, ser activo en tu propio cuidado es una de las mejores maneras de mantenerte saludable.

Si alguien que conoces desea donarte un riñón, esa persona también necesitará pasar por una evaluación de detección para averiguar si es lo suficientemente saludable para donar.

Si es tu hijo quien tiene la enfermedad renal, el trasplante puede ser el mejor tratamiento para él. El trasplante permite que los niños y jóvenes adultos se desarrollen de la manera más normal posible en sus años formativos. Si tu hijo tiene enfermedad renal, querrás considerar seriamente obtener una evaluación de trasplante para él o ella.

Si el proceso de evaluación muestra que un trasplante es adecuado para ti o tu hijo, el siguiente paso es conseguir un riñón adecuado. (Ver "Encontrar un Riñón" a continuación)."


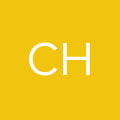


1. **You**

Please translate to Spanish: "What happens during kidney transplant surgery? The operation to place a donated kidney takes about four hours. The donated kidney is placed into your lower abdomen (belly), where it’s easiest to connect it to your important blood vessels and bladder. You may be surprised to learn that your own kidneys generally aren’t taken out when you get a transplant. The surgeon leaves them where they are unless there is a medical reason to remove them. After surgery, you’ll be sore at first, but you should be out of bed in a day or so, and home from the hospital within a week. If the kidney came from a living donor, it should start to work very quickly. A kidney from a deceased donor can take longer to start working—two to four weeks or more. This is called “delayed graft function”. If that happens, you may need dialysis until the kidney begins to work. After surgery, you’ll be taught about the medicines you’ll have to take and their side effects. You’ll also learn about how your diet may change after transplant. If you’ve been on dialysis, you’ll find that there are fewer restrictions on what you can eat and drink, which is one of the benefits of a transplant. You will want to eat a healthy diet and exercise to take good care of your body and your new kidney."

ChatGPT

**ChatGPT**

"¿Qué sucede durante la cirugía de trasplante de riñón? La operación para colocar un riñón donado dura alrededor de cuatro horas. El riñón donado se coloca en tu abdomen inferior (vientre), donde es más fácil conectarlo a tus vasos sanguíneos importantes y a la vejiga. Puede sorprenderte saber que generalmente no se extraen tus propios riñones cuando recibes un trasplante. El cirujano los deja donde están a menos que haya una razón médica para removerlos.

Después de la cirugía, estarás adolorido al principio, pero deberías estar fuera de la cama en un día o dos, y en casa fuera del hospital en aproximadamente una semana. Si el riñón proviene de un donante vivo, debería comenzar a funcionar muy rápidamente. Un riñón de un donante fallecido puede tardar más en empezar a funcionar, de dos a cuatro semanas o más. Esto se llama "función retardada del injerto". Si eso sucede, podrías necesitar diálisis hasta que el riñón comience a funcionar.

Después de la cirugía, se te enseñará sobre los medicamentos que tendrás que tomar y sus efectos secundarios. También aprenderás sobre cómo puede cambiar tu dieta después del trasplante. Si has estado en diálisis, descubrirás que hay menos restricciones en lo que puedes comer y beber, lo cual es uno de los beneficios de un trasplante. Querrás comer una dieta saludable y hacer ejercicio para cuidar bien de tu cuerpo y de tu nuevo riñón."


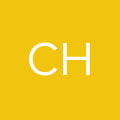


1. **You**

Please translate to Spanish: "What are anti-rejection medicines? Normally, your body fights off anything that isn’t part of itself, like germs and viruses. That system of protection is called your immune system. To stop your body from attacking or rejecting the donated kidney, you will have to take medicines to keep your immune system less active (called anti-rejection medicines or immunosuppressant medicines). You’ll need to take them as long as your new kidney is working. Without them, your immune system would see the donated kidney as “foreign,” and would attack and destroy it. Anti-rejection medicines can have some side effects. It is important to talk to your healthcare provider about them, so that you know what to expect. Fortunately, for most people, side effects are usually manageable. Changing the dose or type of medicine can often ease some of the side effects. Besides the immunosuppressive medicines, you will take other medicines as well. You will take medicines to protect you from infection, too. Most people find taking medicines a small trade for the freedom and quality of life that a successful transplant can provide."

ChatGPT

**ChatGPT**

"¿Qué son los medicamentos anti-rechazo? Normalmente, tu cuerpo combate cualquier cosa que no sea parte de sí mismo, como gérmenes y virus. Ese sistema de protección se llama sistema inmunológico. Para evitar que tu cuerpo ataque o rechace el riñón donado, tendrás que tomar medicamentos para mantener tu sistema inmunológico menos activo (llamados medicamentos anti-rechazo o inmunosupresores). Necesitarás tomarlos mientras tu nuevo riñón esté funcionando. Sin ellos, tu sistema inmunológico vería el riñón donado como “extranjero” y lo atacaría y destruiría.

Los medicamentos anti-rechazo pueden tener algunos efectos secundarios. Es importante hablar con tu proveedor de atención médica sobre ellos, para que sepas qué esperar. Afortunadamente, para la mayoría de las personas, los efectos secundarios suelen ser manejables. Cambiar la dosis o el tipo de medicamento a menudo puede aliviar algunos de los efectos secundarios.

Además de los medicamentos inmunosupresores, también tomarás otros medicamentos. Tomarás medicamentos para protegerte de infecciones, también. La mayoría de las personas consideran que tomar medicamentos es un pequeño precio a pagar por la libertad y calidad de vida que un trasplante exitoso puede proporcionar."


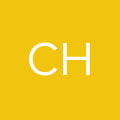


1. **You**

Please translate to Spanish: "Where do donated kidneys come from? A donated kidney may come from someone who died and donated a healthy kidney, called a deceased donor. Donated kidneys also can come from a living donor. This person may be a blood relative (like a brother or sister) or non-blood relative (like a husband or wife). They can also come from a friend or even a stranger. You do not need to have the same blood type as your donor to get a kidney if you are in a paired exchange program. Visit the Programs for Donor/Recipient Pairs with Incompatible Blood Types page to learn more about paired exchange programs. Kidneys from living or deceased donors both work well and can be good options, but kidneys from a living donor may last longer and are more likely to start working right away than a kidney from a deceased donor. Another plus is that when a kidney is donated by a living person, the operations can be scheduled at a convenient time for both the patient and the donor. Finding a living donor can also help you get a kidney faster since waiting for a deceased donor kidney can take many years."

ChatGPT

**ChatGPT**

"¿De dónde provienen los riñones donados? Un riñón donado puede provenir de alguien que falleció y donó un riñón sano, a quien se le llama donante fallecido. Los riñones también pueden provenir de un donante vivo. Esta persona puede ser un familiar de sangre (como un hermano o hermana) o no familiar de sangre (como un esposo o esposa). También pueden provenir de un amigo o incluso de un desconocido. No necesitas tener el mismo tipo de sangre que tu donante para recibir un riñón si estás en un programa de intercambio pareado. Visita la página de Programas para Pares Donante/Receptor con Tipos de Sangre Incompatibles para aprender más sobre los programas de intercambio pareado.

Los riñones de donantes vivos o fallecidos funcionan bien y pueden ser buenas opciones, pero los riñones de un donante vivo pueden durar más y tienen más probabilidades de empezar a funcionar inmediatamente que un riñón de un donante fallecido. Otro punto a favor es que cuando un riñón es donado por una persona viva, las operaciones pueden programarse en un momento conveniente tanto para el paciente como para el donante.

Encontrar un donante vivo también puede ayudarte a conseguir un riñón más rápido, ya que esperar un riñón de un donante fallecido puede llevar muchos años."


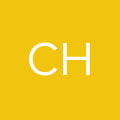


1. **You**

Please translate to Spanish: "How do I get a kidney from a deceased donor? To get a deceased donor kidney, you will be placed on a waiting list once you have been cleared for a transplant. It can take many years for a good donor kidney to be offered to you. From the time you go on the list until a kidney is found, you may have to be on some form of dialysis. While you’re waiting, you’ll need regular blood tests to make sure you are ready when a kidney is found. If you’re on dialysis, your center will make the arrangements for these tests. Your transplant center should know how to reach you at all times. Once a kidney become available, the surgery must be done as soon as possible."

ChatGPT

**ChatGPT**

"¿Cómo obtengo un riñón de un donante fallecido? Para obtener un riñón de un donante fallecido, serás colocado en una lista de espera una vez que hayas sido aprobado para un trasplante. Puede tomar muchos años que se te ofrezca un riñón de un buen donante. Desde el momento en que entras en la lista hasta que se encuentra un riñón, es posible que tengas que estar en alguna forma de diálisis. Mientras esperas, necesitarás análisis de sangre regulares para asegurarte de estar listo cuando se encuentre un riñón. Si estás en diálisis, tu centro hará los arreglos para estas pruebas. Tu centro de trasplante debe saber cómo contactarte en todo momento. Una vez que un riñón esté disponible, la cirugía debe realizarse lo antes posible."


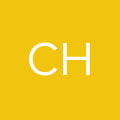


1. **You**

Please translate to Spanish: "How do I get a kidney from a living donor? To receive a kidney from a living donor, you must first be cleared by a transplant center after completing an evaluation. After that, the best place to start is by sharing your story with people you know and your community. Talk to your family and friends about your kidney failure and your need for a donor. To learn more about how to find a living donor, visit this link. "

ChatGPT

**ChatGPT**

"¿Cómo obtengo un riñón de un donante vivo? Para recibir un riñón de un donante vivo, primero debes ser aprobado por un centro de trasplantes después de completar una evaluación. Después de eso, el mejor lugar para comenzar es compartiendo tu historia con las personas que conoces y tu comunidad. Habla con tu familia y amigos sobre tu insuficiencia renal y tu necesidad de un donante.

Para aprender más sobre cómo encontrar un donante vivo, visita este enlace."

1. **You**

Please translate to Spanish: "Are there downsides to living donation? A healthy person who donates a kidney can live a normal life with the one kidney that is left, but a kidney donor must undergo surgery to remove a kidney and give it to the recipient. The operation is major surgery for the donor, as well as the recipient. As in any operation, there are some risks that you will want to consider , including surgery risks and long-term risks. The donor will need time to recover before returning to work and other activities. However, recent advances in surgery (often called minimally invasive or laparoscopic surgery) allow for very small incisions. This means shorter hospital stays and recovery time, less pain, and a quicker return to usual activities. Living donors often experience positive feelings about their courageous gift. Learn more about living donation here. "

ChatGPT

**ChatGPT**

"¿Hay desventajas en la donación en vida? Una persona sana que dona un riñón puede vivir una vida normal con el único riñón que le queda, pero un donante de riñón debe someterse a una cirugía para extraer un riñón y dárselo al receptor. La operación es una cirugía mayor tanto para el donante como para el receptor. Como en cualquier operación, hay algunos riesgos que querrás considerar, incluyendo los riesgos de la cirugía y los riesgos a largo plazo.

El donante necesitará tiempo para recuperarse antes de volver al trabajo y otras actividades. Sin embargo, los avances recientes en cirugía (a menudo llamada cirugía mínimamente invasiva o laparoscópica) permiten incisiones muy pequeñas. Esto significa estadías hospitalarias más cortas y tiempo de recuperación, menos dolor y un regreso más rápido a las actividades habituales. Los donantes vivos a menudo experimentan sentimientos positivos acerca de su valiente regalo. Aprende más sobre la donación en vida aquí."

1. **You**

Please translate to Spanish: "What are the financial costs to the living donor? The surgery and evaluation are covered by Medicare or the recipient’s insurance. The living donor will not pay for anything related to the surgery. However, neither Medicare nor insurance covers time off from work, travel expenses, lodging, or other incidentals. The National Living Donor Assistance Program (www.livingdonorassistance.org) or other programs may help cover travel and lodging costs. Donors may be eligible for sick leave, state disability, and benefits under the federal Family Medical Leave Act. In addition, federal employees, some state employees, and certain other workers may be eligible for 30 days paid leave."

ChatGPT

**ChatGPT**

"¿Cuáles son los costos financieros para el donante vivo? La cirugía y la evaluación están cubiertas por Medicare o por el seguro del receptor. El donante vivo no pagará nada relacionado con la cirugía. Sin embargo, ni Medicare ni los seguros cubren el tiempo libre del trabajo, los gastos de viaje, alojamiento u otros gastos incidentales. El Programa Nacional de Asistencia al Donante Vivo (www.livingdonorassistance.org) u otros programas pueden ayudar a cubrir los costos de viaje y alojamiento.

Los donantes pueden ser elegibles para licencia por enfermedad, discapacidad estatal y beneficios bajo la Ley Federal de Licencia Médica Familiar. Además, los empleados federales, algunos empleados estatales y ciertos otros trabajadores pueden ser elegibles para 30 días de licencia pagada."

1. **You**

Please translate to Spanish: "What if my body tries to reject the new kidney? One thing that you and your healthcare team will watch for is acute rejection, which means that your body is suddenly “attacking” or trying to reject the transplanted kidney. A rejection episode may not have any clear signs or symptoms. That is why it is so important to have regular blood tests to check how well your kidney is working. Even though rejection can happen without symptoms, let your healthcare team know if you notice you are having fevers, peeing less or less often, swelling, weight gain, or pain over your kidney. The chances of having a rejection episode are highest right after your surgery. The longer you have the kidney, the lower the chance that this will happen. Unfortunately, sometimes a rejection episode happens even if you’re doing everything you’re supposed to do. Sometimes the body just doesn’t accept the transplanted kidney. But even if a rejection episode happens, there are many ways to treat it so you do not lose your transplant. Letting your transplant team know right away that you think you have symptoms of rejection is very important so they can treat it and help keep your transplanted kidney, and you, healthy."

ChatGPT

**ChatGPT**

"¿Qué pasa si mi cuerpo intenta rechazar el nuevo riñón? Una cosa que tú y tu equipo de atención médica vigilarán es el rechazo agudo, lo que significa que tu cuerpo está "atacando" o intentando rechazar el riñón trasplantado. Un episodio de rechazo puede no tener signos o síntomas claros. Por eso es tan importante tener análisis de sangre regulares para verificar cómo está funcionando tu riñón. Aunque el rechazo puede ocurrir sin síntomas, informa a tu equipo de atención médica si notas que tienes fiebre, orinas menos o con menos frecuencia, hinchazón, aumento de peso o dolor sobre tu riñón.

Las posibilidades de tener un episodio de rechazo son más altas justo después de tu cirugía. Cuanto más tiempo tengas el riñón, menor será la probabilidad de que esto suceda. Desafortunadamente, a veces un episodio de rechazo sucede incluso si estás haciendo todo lo que se supone que debes hacer. A veces el cuerpo simplemente no acepta el riñón trasplantado. Pero incluso si ocurre un episodio de rechazo, hay muchas maneras de tratarlo para que no pierdas tu trasplante. Informar a tu equipo de trasplante de inmediato si crees que tienes síntomas de rechazo es muy importante para que puedan tratarlo y ayudar a mantener tu riñón trasplantado, y a ti, saludable."

1. **You**

Please translate to Spanish: "How often does rejection happen? Rejections happen much less often than they used to. That’s because there have been many improvements in immunosuppressive medicines. However, the risk of rejection is different for every person. For most people, rejection can be stopped with special anti-rejection medicines. It’s very important to have regular checkups and tests to see how well your kidney is working, and make sure you are not having rejection."

ChatGPT

**ChatGPT**

"¿Con qué frecuencia ocurre el rechazo? Los rechazos ocurren mucho menos a menudo de lo que solían ocurrir. Esto se debe a que ha habido muchas mejoras en los medicamentos inmunosupresores. Sin embargo, el riesgo de rechazo es diferente para cada persona. Para la mayoría de las personas, el rechazo puede detenerse con medicamentos anti-rechazo especiales. Es muy importante tener chequeos y pruebas regulares para ver cómo está funcionando tu riñón y asegurarte de que no estás experimentando rechazo."

1. **You**

Please translate to Spanish: "When can I return to work? How soon you can return to work depends on your recovery, the kind of work you do, and your other medical conditions. Many people can return to work eight weeks or more after their transplant, but sometimes it can be sooner or later. Your transplant team will help you decide when you can go back to work."

ChatGPT

**ChatGPT**

"¿Cuándo puedo regresar al trabajo? Cuándo puedas regresar al trabajo dependerá de tu recuperación, el tipo de trabajo que haces y tus otras condiciones médicas. Muchas personas pueden volver al trabajo ocho semanas o más después de su trasplante, pero a veces puede ser antes o después. Tu equipo de trasplante te ayudará a decidir cuándo puedes volver al trabajo."

1. **You**

Please translate to Spanish: "How will a transplant affect my sex life? Can I still have children? People who have not had satisfactory sexual relations due to kidney disease may notice an improvement as they begin to feel better. In addition, fertility (the ability to conceive and have children) tends to increase. Men who have had a kidney transplant have fathered healthy children, and women with kidney transplants have had successful pregnancies. Talk to your healthcare practitioner when considering having a child. Women should avoid becoming pregnant too soon after a transplant. Most centers want women to wait a year or more. All pregnancies should be planned together with your care team. Certain medications can harm a developing baby, so they must be stopped at least six weeks before trying to get pregnant. Birth control or family planning counseling may be helpful. It’s important to protect yourself against sexually transmitted diseases (STDs). Be sure to use protection during sexual activity."

ChatGPT

**ChatGPT**

"¿Cómo afectará un trasplante mi vida sexual? ¿Puedo tener hijos? Las personas que no han tenido relaciones sexuales satisfactorias debido a la enfermedad renal pueden notar una mejora a medida que comienzan a sentirse mejor. Además, la fertilidad (la capacidad de concebir y tener hijos) tiende a aumentar. Hombres que han recibido un trasplante de riñón han engendrado hijos sanos, y mujeres con trasplantes de riñón han tenido embarazos exitosos. Habla con tu profesional de atención médica cuando estés considerando tener un hijo.

Las mujeres deben evitar quedar embarazadas demasiado pronto después de un trasplante. La mayoría de los centros quieren que las mujeres esperen un año o más. Todos los embarazos deben planificarse junto con tu equipo de atención. Ciertos medicamentos pueden dañar a un bebé en desarrollo, por lo que deben suspenderse al menos seis semanas antes de intentar quedar embarazada. La asesoría en control de natalidad o planificación familiar puede ser útil.

Es importante protegerse contra las enfermedades de transmisión sexual (ETS). Asegúrate de usar protección durante la actividad sexual."

1. **You**

Please translate to Spanish: "Will I need to follow a special diet? In general, transplant recipients should eat a heart-healthy diet (low fat, low salt) and drink plenty of fluids. If you have diabetes or other health problems, you may still have some dietary restrictions. A dietitian can help you plan meals that are right for you."

ChatGPT

**ChatGPT**

"¿Necesitaré seguir una dieta especial? En general, los receptores de trasplantes deben comer una dieta saludable para el corazón (baja en grasas, baja en sal) y beber abundantes líquidos. Si tienes diabetes u otros problemas de salud, es posible que aún tengas algunas restricciones dietéticas. Un dietista puede ayudarte a planificar comidas adecuadas para ti."

1. **You**

Please translate to Spanish: "What else can I do? You should learn as much as you can by reading and talking to your healthcare team, as well as patients who already have kidney transplants. To obtain a free copy of a brochure about kidney transplant, call 855.NKF.CARES (855.653.2273) or email nkfcares@kidney.org and ask for “Kidney Transplant: What You Need to Know.”"

ChatGPT

**ChatGPT**

"¿Qué más puedo hacer? Deberías aprender todo lo que puedas leyendo y hablando con tu equipo de atención médica, así como con pacientes que ya tienen trasplantes de riñón.

Para obtener una copia gratuita de un folleto sobre el trasplante de riñón, llama al 855.NKF.CARES (855.653.2273) o envía un correo electrónico a nkfcares@kidney.org y pide "Trasplante de Riñón: Lo Que Necesitas Saber"."
